# Design, Development, and Laboratory Validation of a Low-Cost Multi-Sensor System for Bridge Structural Health Monitoring with Scour-Related Environmental Sensing

**DOI:** 10.3390/s26185957

**Published:** 2026-09-20

**Authors:** Matilde Bidone, Mauro Aimar, Marco Civera, Alessio Carullo, Alberto Vallan

**Affiliations:** 1 Department of Structural, Geotechnical and Building Engineering (DISEG), Politecnico di Torino, Corso Duca degli Abruzzi 24, 10129 Turin, Italy; matilde.bidone@studenti.polito.it (M.B.); mauro.aimar@polito.it (M.A.); 2 Department of Electronics and Telecommunications (DET), Politecnico di Torino, Corso Duca degli Abruzzi 24, 10129 Turin, Italy; alessio.carullo@polito.it (A.C.); alberto.vallan@polito.it (A.V.)

**Keywords:** structural health monitoring, low-cost sensor network, MEMS accelerometers, multi-sensor data acquisition, operational modal analysis, environmental sensing, bridge scour

## Abstract

**Highlights:**

- A low-cost wired multi-sensor platform is designed and prototyped for bridge Structural Health Monitoring with complementary scour-related environmental sensing, combining structural nodes for vibration and rotation monitoring with one environmental node for wind, rainfall, air temperature, humidity and water-level acquisition.
- Laboratory validation confirms reliable communication and acquisition. The prototyped system recovered the imposed shaker frequencies, identified the first six natural frequencies of a scaled structural benchmark, showed coherent trends relative to reference accelerometers and correctly processed simulated environmental sensor inputs.
- The proposed sensing apparatus provides a scalable, cost-conscious acquisition layer for bridge SHM and environmental/hydrological contextual monitoring, with hardware procurement costs of about €1800 for the laboratory prototype and about €4100 for a representative multi-span configuration, excluding VAT and installation/lifecycle costs.
- By integrating structural and environmental measurements in a single synchronised architecture, the platform provides a practical basis for future field deployment, such as finite element model updating and automated damage-detection algorithms, useful for both academic research and practical applications.

**Abstract:**

This paper reports the design, development and laboratory validation of a low-cost multi-sensor system for bridge Structural Health Monitoring with complementary scour-related environmental sensing. The proposed sensing apparatus is intended for continuous dynamic (vibration-based) and quasi-static structural monitoring, in particular for bridge applications. It employs a distributed wired architecture comprising two custom printed circuit boards, BridgeWatch and EnvironMonitor. BridgeWatch nodes integrate MEMS accelerometers and gyroscopes, enabling acceleration measurements, quasi-static inclination estimates from gravity components, angular-rate measurements and structural surface-temperature acquisition. The EnvironMonitor node acquires wind, rainfall, air temperature, humidity and bridge water level; these variables provide environmental and hydrological context relevant to scour risk, without directly measuring riverbed elevation or scour depth. A Raspberry Pi coordinates acquisition, synchronisation and communication through RS485 and RS232 links. The hardware was implemented through custom PCB design and dedicated firmware. The principal contribution is the system-level integration of structural and environmental measurements in one open architecture. Laboratory validation comprised communication and synchronisation checks, shaker tests, a scaled structural benchmark, climatic-cell tests and environmental-input simulations. The results demonstrate functional acquisition and relevant dynamic-feature extraction under controlled conditions. These outcomes enable future full-scale in situ validation and testing on full-size case studies.

## 1. Introduction

Currently, several threats affect the long-term structural integrity and safety of existing bridges. Among them, one can recall material ageing and degradation (including corrosion) [1], increasing traffic demand and extreme environmental actions (including hydrological hazards such as floods and scour). In countries with extensive bridge stocks, many structures were built decades ago under design assumptions and durability requirements that differ from present operating conditions. Hence, the current situation is one of low but consistent bridge failure risk; for instance, it is estimated that road bridges in Italy have a yearly failure rate of $5\times 10^{-5}$ ($2\cdot 10^{-5}$ for rail bridges) [2,3]. In this context, continuous Structural Health Monitoring (SHM) has therefore become an increasingly relevant complement to periodic visual inspection because it provides time-resolved measurements of a structure’s response and the environmental actions acting on it [4].

Despite the availability of mature sensing technologies, widespread permanent monitoring of ordinary small- and medium-sized bridges remains constrained by instrumentation cost, installation complexity, power and communication requirements, and the need to deploy several sensing modalities simultaneously. This is particularly relevant for water-crossing bridges, where structural response cannot be interpreted independently of environmental and hydrological conditions, e.g., rainfall and water level. Deploying independent proprietary systems for different physical variables increases cost and integration complexity. The engineering motivation of the present work is therefore to place these quantities within a common, modular and economically deployable acquisition architecture.

In more detail, SHM can be broadly categorised according to the specific physical quantity measured and used as a damage proxy. Static monitoring uses features that reflect the static (or quasi-static) behaviour of the target structure, such as deviations from verticality in column elements. Conversely, vibration-based SHM is grounded in the relationship between a structure’s physical properties and its modal parameters. Changes in stiffness, mass distribution, constraints or damage state can modify natural frequencies, mode shapes and damping ratios [5]. For bridges, these changes may result from deterioration of structural members, modifications to bearings or supports, loosening of connections or changes in foundation conditions, including scour-induced effects. In particular, this last point is a major issue, as scour has been identified as a major contributor to bridge failures, accounting for up to an estimated ∼80% of all bridges’ total or partial collapses in a recent inventory of Italian infrastructures [2]. Although reported statistics depend strongly on the region, bridge stock and other factors, scour remains one of the most prominent collapse-related hazards for water-crossing bridges and viaducts.

In this context, it is important to note that both static and dynamic monitoring have strengths and weaknesses. It has been widely shown that different damage-sensitive features respond more or less efficiently to different damaging phenomena and collapse mechanisms. As a few examples, mode shapes outperform natural frequencies for localised damage, and different natural frequencies are differently responsive to specific damage locations—for example, in the famous Z24 case study [6,7,8,9], the induced pier damage was more visible in the first lateral mode, due to the prominence of pier stiffness (or lack thereof) in its free vibrations [10]. Instead, in cases where the bridge deck is in good structural condition, e.g., because of recent retrofitting, the principal risk may be associated with pier rotations caused by scour [11]. In such instances, static (rotation-based) monitoring alone makes more economic sense, in terms of cost-effectiveness and value of information (as quantitatively demonstrated in [12]); such an example can be found in the recently instrumented Amedeo VIII Bridge in Turin [13].

For all these reasons, the general consensus is that both static and dynamic features should be collected and analysed to gain a holistic and global vision of the target structure. Furthermore, environmental and operational variabilities [14] should be included in the data processing and interpretation process as well. These act as both confounding influences (e.g., temperature-induced stiffness changes, which cause damage-unrelated shifts in the natural frequencies [15,16]) and as indicators of possible collapse-triggering events. For instance, scour-related phenomena are strongly influenced by river-water level, rainfall and flow-related environmental conditions. Thus, a monitoring system that combines vibration measurements with meteorological and hydrological observations is better suited to distinguishing structural changes from environmental effects and to supporting subsequent damage-detection routines.

The multi-sensor hardware system proposed and presented in this paper follows this logic, informed by these considerations. This apparatus was developed within the *AMBROSE—A Multisensor BRidge MOnitoring SystEm* project [17] to produce a low-cost, modular, and remotely accessible acquisition platform for direct bridge monitoring.

### State of the Art in Low-Cost and Prototype Sensor Systems for SHM

The recent literature shows a clear shift from isolated instrumentation to deployable prototype systems that integrate sensing, local acquisition, synchronisation, communication and power management functions. This trend is particularly relevant for bridges, where long-term monitoring must remain affordable, robust, and scalable across multiple spans and difficult-to-access locations. Several studies have therefore focused not only on the sensing principle itself but also on the complete acquisition chain (including microcontrollers, embedded storage, communication protocols, edge processing and field packaging).

Among vibration-based-only approaches, Muttillo et al. [18] proposed an IoT-oriented SHM system comprising customised triaxial accelerometer nodes and a datalogger capable of synchronising acquisitions and communicating via RS485; the system was tested on a laboratory structure and used to compute a structural damage indicator. Zanelli et al. [19] developed and field-validated a prototype named WindNode, a low-power wireless accelerometric node designed for continuous monitoring; the prototype combines MEMS acceleration sensing, onboard FFT processing, Bluetooth Low Energy communication and energy harvesting for autonomous operation. Villacorta et al. [20] presented a scalable, reconfigurable, low-cost system based on MEMS triaxial accelerometers, a myRIO acquisition platform and LabVIEW-based processing, showing good agreement with a conventional piezoelectric accelerometer system in modal tests.

Bridge-specific low-cost prototypes have also been reported. Komarizadehasl et al. [21] introduced LARA, a Low-cost Adaptable Reliable Accelerometer based on Arduino technology, reporting a noise density of 51 µg/√Hz, and validated it on a short-span footbridge against a commercial high-precision sensor. The same research line later demonstrated operational and analytical modal analysis of a bridge using low-cost wireless Arduino-based accelerometers [22]. Hasani et al. [23] proposed a battery-powered wireless MEMS data acquisition system for bridge operational modal analysis, including preprocessing, synchronisation, web-based configuration and model-structure validation; the method was field-tested with a 30-sensor installation on a concrete arch bridge. Hidalgo-Fort et al. [24] developed a low-cost, low-power IoT monitoring node with modular hardware and edge computing capabilities for bridge SHM, integrating local processing to reduce wireless data transmission. Di Nuzzo et al. [25] proposed a MEMS-based wireless node using Narrowband IoT communication, targeting long-term modal monitoring with low power consumption and energy-neutral operation through a small solar panel.

Other sensing prototypes address complementary, non-dynamic SHM quantities in addition to acceleration. For instance, Park et al. [26] developed a wireless laser displacement sensor node using a laser displacement sensor, a customised low-power node and CDMA communication for long-distance field deployment. Feng et al. [27] proposed a non-contact, vision-based displacement sensor using subpixel template matching, which was validated in shaking-table tests and bridge field measurements. Aygun et al. [28] produced a large-area resistive strain-sensing sheet using flexible PCB technology, enabling distributed strain sensing and crack-growth tracking in laboratory and field structures. Pham et al. [29] designed a PZT-embedded smart rock for electromechanical-impedance monitoring of internal concrete damage in a prestressed anchorage zone. As one last example, Li et al. [30] designed and tested an FMCW radar prototype for non-contact displacement measurement and multi-target identification in civil infrastructure monitoring.

Smartphone-based sensing represents a complementary low-cost paradigm for bridge monitoring. Recent studies from Feng et al. [31,32], the App4SHM work of Figueiredo et al. [33] and the review work by Ozer and Kromanis [34] show that embedded smartphone accelerometers can support bridge-vibration measurements and modal identification at negligible additional sensor cost. Their use for permanent SHM, however, raises issues associated with heterogeneous sensor fidelity, clock stability and synchronisation, device orientation and mounting, and unattended long-term operation. The architecture developed here addresses a different monitoring scenario: fixed, persistent and multi-point instrumentation in which the sensing hardware, sampling strategy, communication architecture and environmental measurements are defined and controlled by the monitoring-system designer.

Researchers have also developed complementary low-cabling solutions for strain-based SHM. Liu et al. [35] reviewed passive, active, semi-passive, UHF, chipless and multi-sensory RFID strain-sensing approaches, while Wang et al. [36] proposed a dual-interrogation-mode RFID strain sensor that combines passive, low-power operation with active, long-distance interrogation. At the subsequent interpretation level, recent research increasingly relies on probabilistic and machine-learning approaches to distinguish structural changes from normal variability; for example, Wang et al. [37] proposed a sparse-Bayesian-learning damage-detection framework using healthy-state monitoring data. These developments reinforce the need for reliable and configurable sensing hardware capable of supplying subsequent model-based and data-driven SHM procedures.

These examples show that effective prototype SHM systems are increasingly multi-functional: they combine low-cost or miniaturised sensing, especially MEMS-based accelerometers (thanks to all their technical and practical advantages [38]), embedded acquisition, synchronisation, communication, and in several cases, onboard or edge data reduction. The system proposed in this paper follows this same direction, but with a specific emphasis on a low-cost architecture that jointly acquires structural vibration data and environmental and scour-related variables. Unlike many vibration-only prototypes, the proposed design explicitly includes wind, rain, air temperature, humidity, structural temperature and bridge water-level inputs, enabling physical interpretation of modal-response variations alongside environmental loading and hydraulic boundary conditions.

The cited architectures also expose recurring trade-offs. Vibration-only systems can provide high-quality modal information but do not directly supply the environmental and hydrological context required to interpret long-term changes. Wireless and NB-IoT nodes reduce cabling, but synchronisation, radio energy demand and communication availability become central design constraints. Conversely, offline local-storage systems can achieve excellent autonomy and timing stability but sacrifice continuous remote visibility. Smartphone approaches minimise dedicated hardware cost but offer less control over sensor fidelity, mounting and timing. Thus, no single topology simultaneously maximises sensing breadth, timing performance, autonomy, remote accessibility and installation simplicity; the appropriate architecture depends on the monitoring objective.

In this framework, the novelty proposed in this paper does not reside in any individual MEMS sensor, Raspberry Pi computer or standard communication protocol, nor in introducing a new sensing principle. Rather, the proposed design provides a configurable multi-domain acquisition system, and the main contributions lie in the following:

(1) The co-design of structural and environmental/hydrological acquisition within one configurable architecture, with system-level integration;
(2) The common wired data-and-power backbone for multiple structural nodes;
(3) The combined support for vibration, quasi-static structural response, structural temperature and environmental context; and
(4) The PCB/firmware implementation whose individual acquisition functions are checked under controlled inputs.

Notably, a recent paper by Alandí et al. [39] reports an offline, synchronised multi-sensor DAQ architecture for bridge SHM. Some component choices overlap with the present work (most notably the ADXL355 accelerometer and STM32L431 microcontroller) as both designs target low-amplitude civil-structure measurements using commercially available low-noise MEMS technology. However, the underlying systems differ in terms of sensing devices, as well as storage, synchronisation, connectivity and system powering. The hardware described in the present study was also designed in 2024, before publication of [39].

The remainder of this paper is organised as follows. Section 2 details the design objectives and monitoring requirements. Section 3, Section 4 and Section 5 report, in the same order, the system architecture, the hardware design, and the communication architecture and firmware. Section 6 describes the laboratory experimental validation; Section 7 reports on power-consumption estimates, scalability and cost analysis, while Section 8 briefly discusses mechanical integration for future outdoor deployment. Discussion and conclusions are reported in Section 9 and Section 10, respectively.

This study intentionally focuses on the design, implementation and validation of the hardware architecture under known, controlled laboratory conditions. In situ tests on full-scale bridge case studies constitute the immediate next validation stage and will be followed up in dedicated future work.

## 2. Design Objectives and Monitoring Requirements

The system’s target application is intentionally limited to the continuous monitoring of small- to medium-sized water-crossing bridges, with particular attention to representative multi-span configurations. Therefore, the hardware design was driven by six requirements:

1. The use of sensors suitable for vibration-based SHM and environmental monitoring;
2. Sufficient sampling frequency for bridge dynamic response;
3. Reliable wired communication over outdoor cable runs;
4. Modularity, so that the number of structural nodes can be adapted to the bridge geometry;
5. Minimally intrusive installation at sensor locations, avoiding drilling or permanent alteration of the bridge where possible while allowing routed power/data cabling; and
6. Low cost with autonomous power capability.

The structural node must measure low-amplitude accelerations in the frequency range relevant to bridge dynamics, while also providing angular rate and surface temperature information. To this end, a sampling frequency of 256 Hz was selected (the selected ADXL355 sensor allows a configurable output data rate of up to 4 kHz). This value is higher than the minimum required for the first modal frequencies of many concrete bridges, while providing adequate bandwidth for laboratory validation. It also leaves a margin for higher-frequency structural components and transient events. In addition, based on previous ambient vibration tests (AVTs) recently performed in a wide array of bridges with different structural configurations and building materials, amplitude-resolution and sensitivity requirements were also formulated, aiming at capturing AVT signals as low as 10 mg (1 thousandth of g, where $g=9.806\;m/s^{2}$) in peak-to-peak amplitude. In this sense, in the ±2 g range, the ADXL355 sensor provides a digital resolution of 3.9 µg/LSB and a noise density of 22.5 µg/√Hz, making acceleration amplitudes on the order of ±0.01 g in the vertical direction (and even smaller horizontal components) measurable under suitable signal-to-noise ratio (SNR) conditions with adequate resolution. Please note that the quoted 22.5 µg/√Hz value is the sensor datasheet noise density and is used here for component selection; the present campaign did not experimentally establish an assembled-node minimum detectable acceleration or tilt.

Importantly, in addition to the ADXL355 accelerometer, each BridgeWatch node integrates an I3G4250D three-axis MEMS gyroscope, which provides direct angular-rate measurements and complementary information for rotation-related monitoring. Concerning rotations, it is therefore important to distinguish between quasi-static inclination estimates and angular-rate measurements.

Thus, the ADXL355 MEMS accelerometer integrated in BridgeWatch can be used to estimate quasi-static inclination, because it measures the gravity vector and has a response extending down to 0 Hz. After suitable low-pass filtering, the inclination can be obtained from the relative gravity components measured along the sensor axes. Conversely, the I3G4250D gyroscope measures angular velocity rather than absolute rotation, and dynamic rotations must therefore be estimated by time integration.

This distinction has important consequences for drift. For small inclinations, the angular error associated with an accelerometer offset can be approximated as $\delta \theta_{acc}\approx \delta a/g$. The ADXL355 datasheet specifies a maximum zero-g offset temperature coefficient of 0.15 mg/°C, which corresponds to an approximate maximum inclination variation of $1.5\cdot 10^{-4}$ rad/°C, or 0.0086°/°C (8.6 mdeg/°C), close to the horizontal condition. This error does not increase indefinitely with measurement duration, although accelerometer-based inclination remains affected by initial offset, noise, cross-axis sensitivity, temperature and mounting conditions.

By comparison, the I3G4250D datasheet reports a zero-rate-level change with temperature of approximately ±0.03 degrees per second (dps) per °C in the ±245 dps range. If this residual angular-rate bias is not compensated, the resulting integrated angular error can be approximated as $\delta \theta_{gyro}\approx 0.03\Delta Tt$, with the angle expressed in degrees and time in seconds. Thus, a bias variation associated with a temperature change of only 1 °C would theoretically produce an angular error of approximately 0.03° after 1 s and 1.8° after 60 s. The gyroscope sensitivity of 8.75 mdps/LSB corresponds to a nominal angular increment of approximately 0.0342 mdeg over one sampling interval at 256 Hz; however, this quantisation value should not be interpreted as the achievable accuracy of an integrated rotation estimate.

Therefore, accelerometer-based inclination is more appropriate for static and slowly varying bridge rotations, whereas gyroscope integration is restricted primarily to short-duration dynamic rotations unless dedicated bias estimation, temperature compensation or other procedures (e.g., data fusion) are applied. However, this study did not include controlled tilt calibration or accelerometer–gyroscope fusion experiments, as they were beyond the intended aims of this research. Therefore, quasi-static inclination and short-duration angular-rate sensing are treated as hardware capabilities of BridgeWatch, whereas high-precision fused inclination is reserved for future, dedicated studies.

Finally, the environmental node had to collect quantities that influence structural response: wind speed and direction, rain rate, air temperature, relative humidity and river water level. In this configuration, scour is not measured directly as a bed-elevation change; rather, water-level measurements and rainfall/wind data provide contextual variables relevant to interpreting bridge-scour risk and to future correlation with structural response. However, with the exception of specific applications such as very slender suspension bridges, where wind speed must be quantified with high accuracy [40], the sensing requirements for the remaining environmental quantities generally fall within the performance range of low-cost sensing devices. Consequently, these measurements did not pose a major design challenge, and all selected components, described in detail in the following sections, met the expected system-level requirements.

## 3. System Architecture

**Figure 1:**
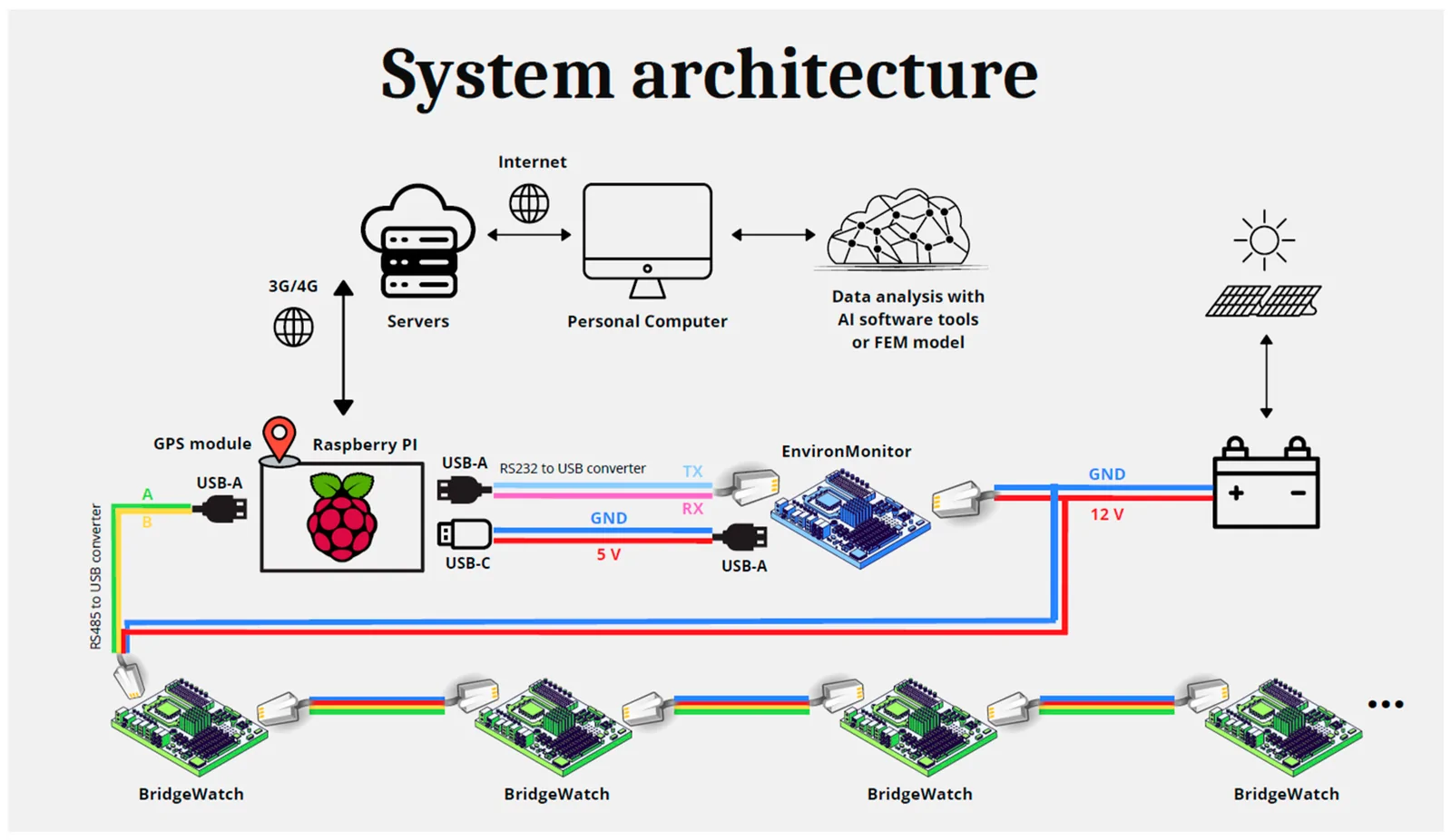
Overall architecture of the proposed multi-sensor bridge monitoring system, including the Raspberry Pi coordinator, the RS485 structural bus, the RS232 environmental link, remote access, server-side storage and autonomous power supply.

The complete system is based on a distributed wired architecture, shown in Figure 1. Multiple BridgeWatch boards are deployed along the structure (ideally at piers and along the spans), while one EnvironMonitor board is installed in a location suitable for environmental and hydrological sensing. A Raspberry Pi acts as the local coordinator. It communicates with the BridgeWatch nodes through an RS485 bus and with the EnvironMonitor node through an RS232 link. A 12 V battery recharged by photovoltaic panels supplies the field system. The wired topology was deliberately selected to favour deterministic, error-checked communication and a shared central power source, avoiding a separate battery and radio at each structural node. Accordingly, the wired architecture was preferred to a wireless (cable-free) installation. The trade-off is the practical burden of routing and protecting the RS485/power cable, which will be evaluated in the planned field deployment.

The Raspberry Pi also manages the GPS module for time and global positioning data, creates acquisition files and is intended to forward data to a remote server via a cellular connection. In the tested prototype, the Raspberry Pi is primarily responsible for acquisition coordination, communication management, time organisation, parsing and local storage of the raw structural and environmental measurements. The signal-processing routines used for the laboratory validation, including FFT-based frequency extraction and modal analysis, as described in the following sections, were executed offline on a laboratory computer after data acquisition. The current prototype presented here does not implement or validate any on-board FFT, SSI or damage-identification routine. Nevertheless, the proposed architecture was conceived to support future edge-computing implementation, in which selected processing routines can be executed directly on the Raspberry Pi before data transmission.

This topology separates high-rate structural measurements from lower-rate environmental quantities, while preserving a common time base. BridgeWatch files include temperature, three-axis acceleration, accelerometer temperature and three-axis angular velocity for each structural node. The EnvironMonitor file contains elapsed time, wind speed, wind direction, rainfall rate, water level, air temperature and relative humidity. The file structure is intentionally simple, with header and unit rows followed by semicolon-separated measurements, so that standard numerical tools can process the data directly. To keep the discussion here relatively short, the full schematics and main circuit blocks are reported in Supplementary Materials (Section A, Figures S1–S5).

## 4. Hardware Design

**Figure 2:**
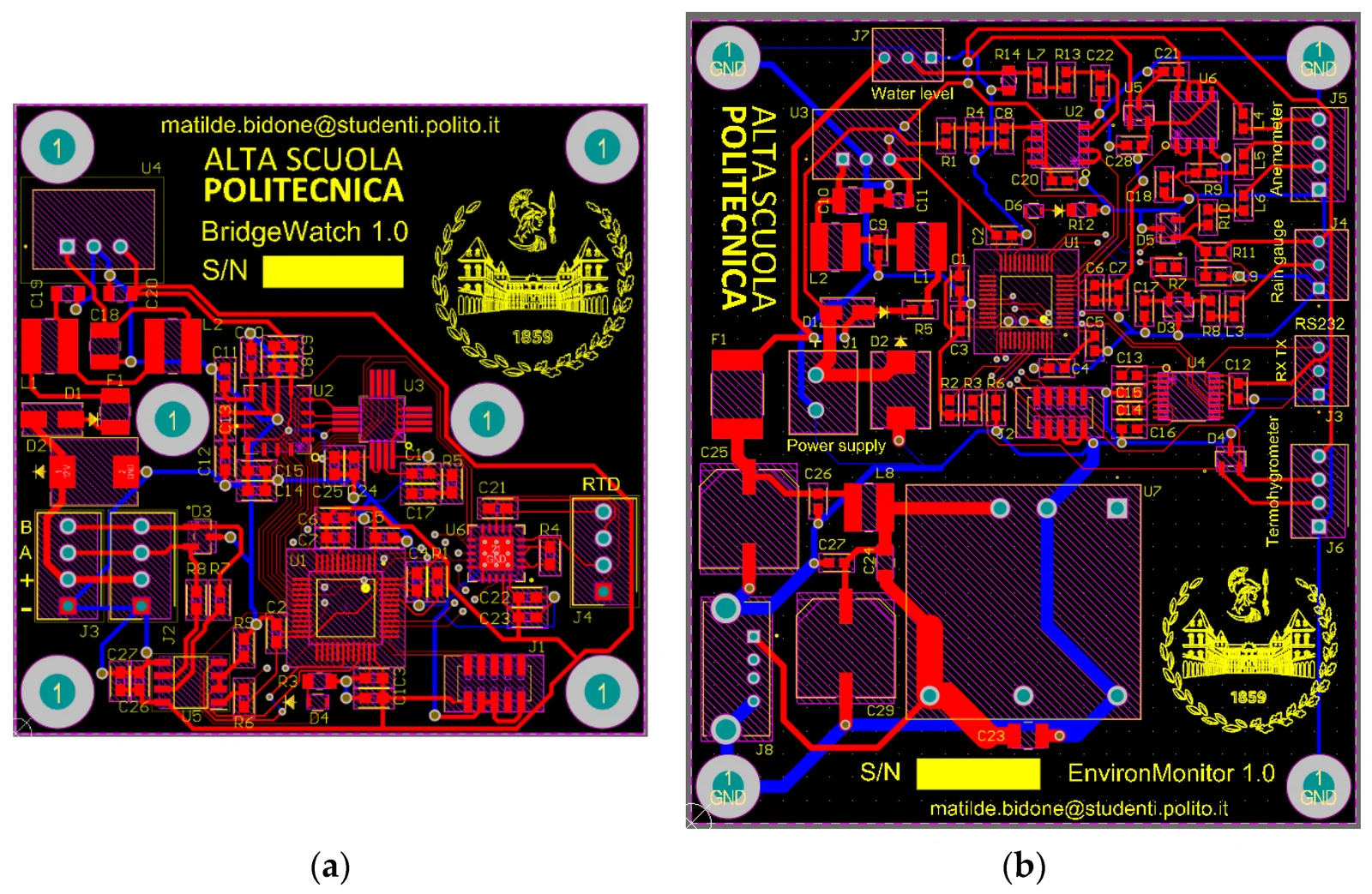
(**a**) BridgeWatch PCB layout, with 6 cm × 6 cm board dimensions and the main structural-sensing and communication blocks. (**b**) EnvironMonitor PCB layout, with 7.2 cm × 8.7 cm board dimensions and connectors for wind, rainfall, air temperature, humidity and water-level measurements.

In the following subsections, the key aspects of the BridgeWatch Structural Node (printed circuit board, or PCB, layout shown in Figure 2a) and the EnvironMonitor Environmental Node (Figure 2b) are reported and discussed in depth.

### 4.1. BridgeWatch Structural Node

As already hinted before, BridgeWatch integrates a low-noise ADXL355 MEMS accelerometer and an I3G4250D MEMS gyroscope. These are coupled with a PT100 structural temperature sensor, a MAX31865 RTD-to-digital converter, an STM32L431CBT6 microcontroller, a 12 V-to-3.3 V DC−DC converter, an RS485 transceiver, a JTAG/SWD programming connector, a status LED and connectors for the shared data-power bus. The board layout is shown in Figure 2a.

The ADXL355 was selected for the reasons already explained in Section 2, most importantly for its low noise, low drift, digital output and suitability for both static and dynamic measurements. Its measurement range can be configured to ±2 g, ±4 g, or ±8 g, with a resolution compatible with low-amplitude vibration acquisition. The gyroscope provides short-duration angular-rate information, whereas quasi-static inclination is primarily estimated from the gravity components measured by the accelerometer (as before, per the requirements highlighted in Section 2). The PT100 interface enables local measurement of structural surface temperature, which matters because modal parameters and sensor response can vary with temperature.

### 4.2. EnvironMonitor Environmental Node

EnvironMonitor is the environmental acquisition node. As already shown in Figure 2b, the system integrates connectors and signal-conditioning circuits for a VEGAPULS C 23 water-level sensor by VEGA, a SKU 7911 anemometer and a SKU 6464 rain gauge by Davis Instruments, and an AM2315C thermohygrometer by Asair. It also contains the microcontroller, an RS232 transceiver, a 12 V-to-3.3 V converter for the board electronics, a 12 V-to-5 V converter for the Raspberry Pi supply, a voltage reference for the ADC, operational amplifier buffers and battery voltage monitoring. The environmental sensors were selected to provide direct measurements of external variables that may affect the bridge response or the boundary conditions at the foundations. The water-level measurement is based on a radar level sensor with 4–20 mA output, which is converted to a voltage suitable for the microcontroller ADC. The rain gauge is a tipping-spoon device generating a switch closure for each 0.2 mm of rainfall. The anemometer provides wind-speed pulses and wind-direction information through a potentiometric output. The thermohygrometer communicates via I2C and provides calibrated temperature and humidity data.

### 4.3. PCB Implementation and Assembly

**Figure 3:**
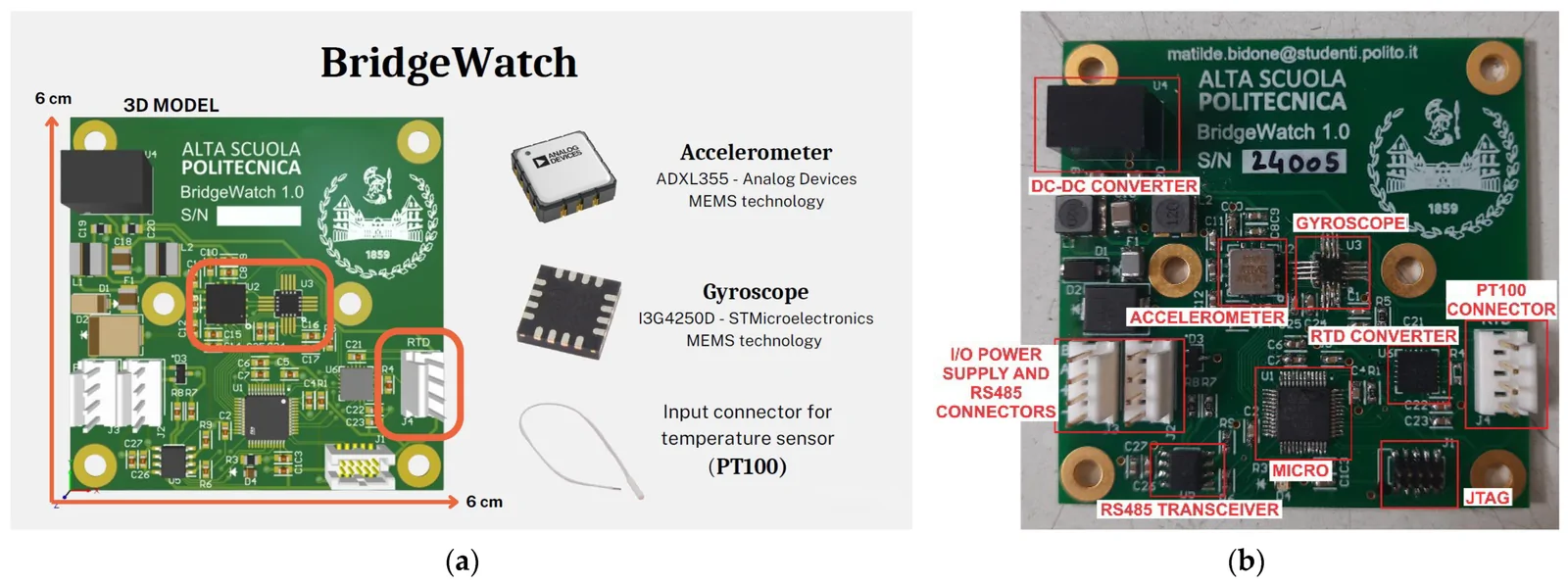
BridgeWatch PCB: (**a**) 3D CAD view, highlighting the ADXL355 accelerometer, I3G4250D gyroscope and PT100 input connector; (**b**) actual picture after component soldering, with the main components and connectors labelled.

After the schematic and layout stages, the two boards were produced from Gerber files and manually assembled. Machine soldering was not adopted in the prototype phase; hence, manual assembly required careful handling of the small surface-mount components and microscope-assisted soldering for fine-pitch packages. Figure 3a presents the BridgeWatch 3D CAD view, where the accelerometer, gyroscope and PT100 input connector are identified and highlighted. Figure 3b reports an actual picture of the BridgeWatch board after soldering, also identifying the main components on the assembled board. The final board includes the DC−DC converter, accelerometer, gyroscope, RTD converter, microcontroller, RS485 transceiver, JTAG connector and the connectors used for power and communication.

**Figure 4:**
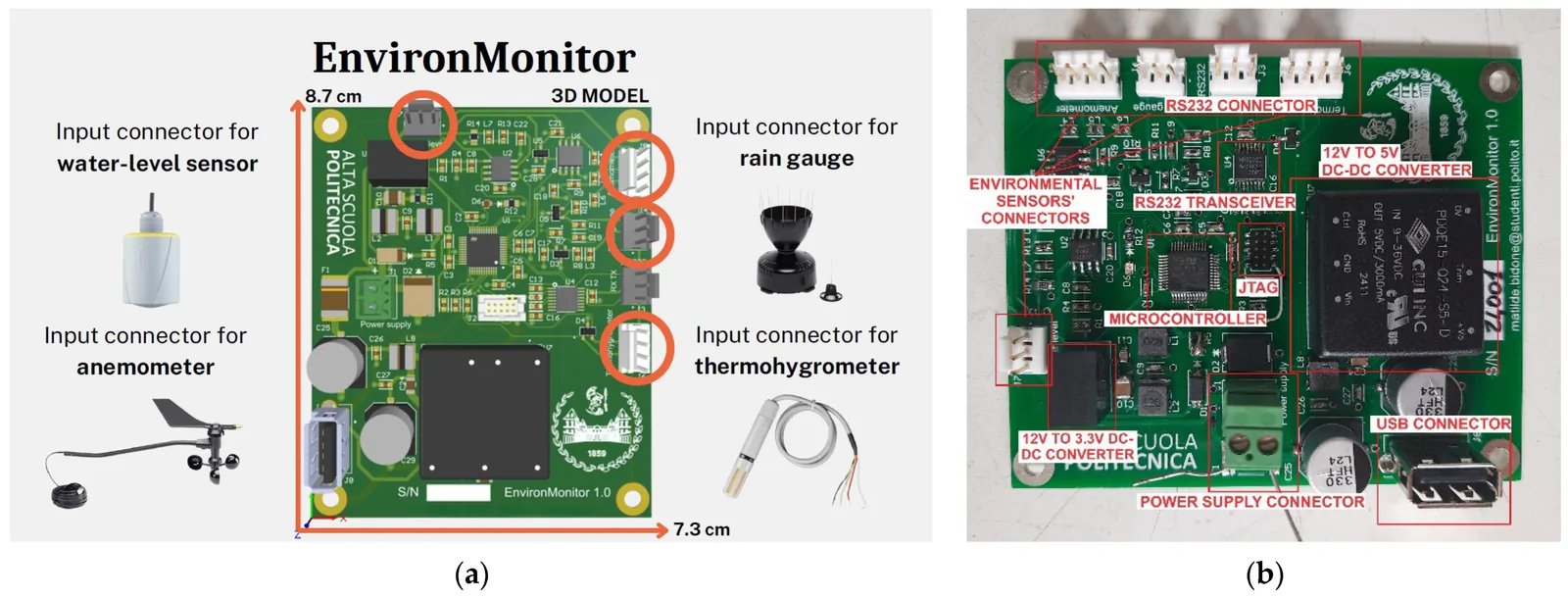
EnvironMonitor PCB: (**a**) 3D CAD view, highlighting the connectors assigned to the water-level sensor, anemometer, rain gauge and thermohygrometer; (**b**) actual picture after component soldering, with the main functional blocks and connectors labelled.

The EnvironMonitor board follows the same design and assembly process. Figure 4a shows the 3D CAD model and highlights the environmental sensor connectors, while Figure 4b portrays the board after soldering, also labelled with the main components, including the RS232 transceiver, DC−DC converters, microcontroller, sensor input connectors, voltage reference and power connector.

## 5. Communication Architecture and Firmware

**Figure 5:**
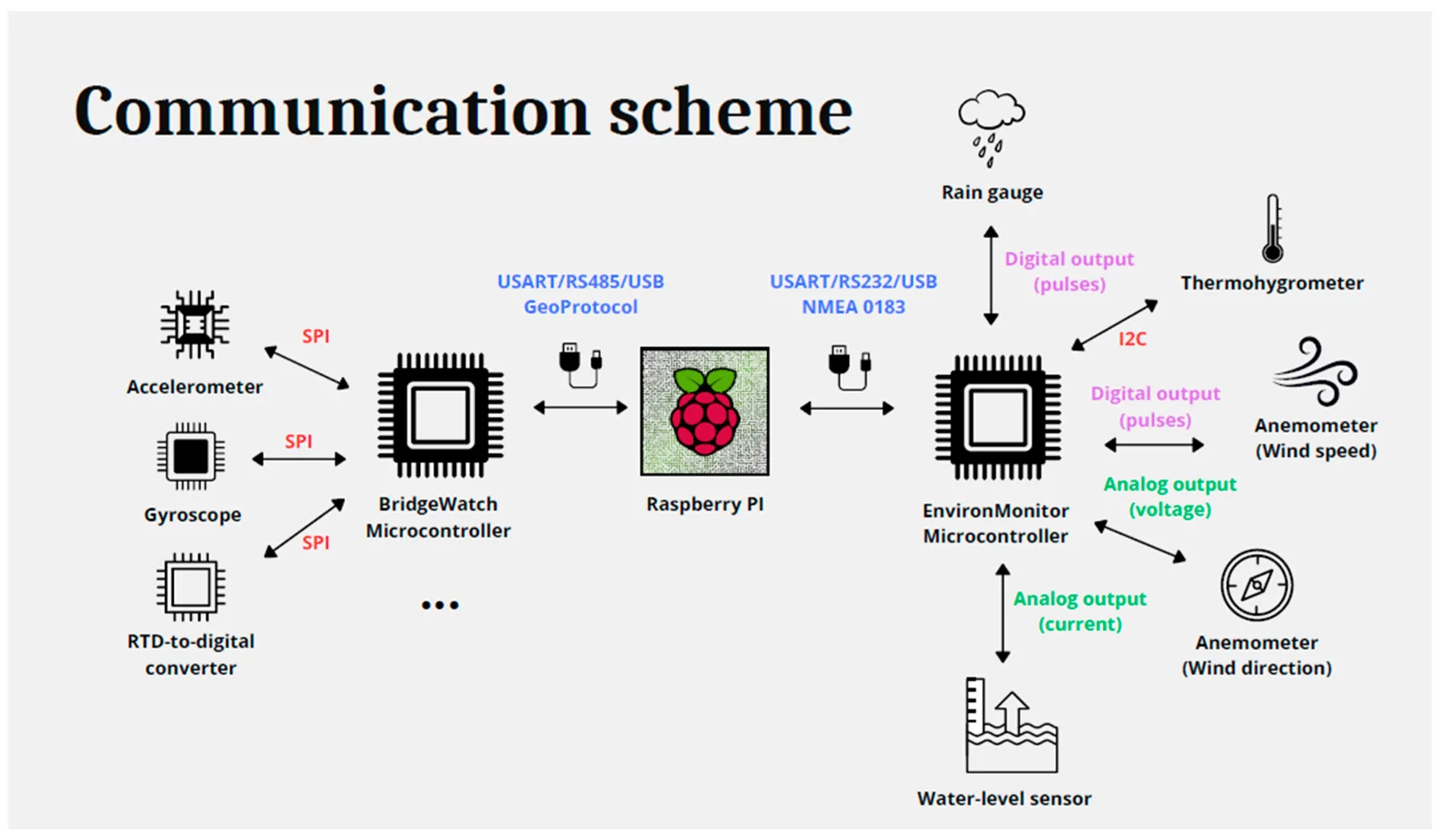
Communication scheme and protocols used in the proposed system: SPI on BridgeWatch, I2C and analogue/digital inputs on EnvironMonitor, RS485 for the structural bus and RS232/NMEA-style communication for environmental data.

Data transmission is essential in SHM, as assessment quality depends on the quality of the processed data, which is derived from the entire measurement chain rather than solely from the raw reading at the single-transducer level. Figure 5 summarises the communication architecture. Communication inside the single boards relies on short-range digital protocols and analogue acquisition: BridgeWatch uses SPI for the accelerometer, gyroscope and RTD converter; EnvironMonitor uses I2C for the thermohygrometer, ADC channels for analogue outputs and interrupt/counter logic for pulse-based sensors. Long-distance communication is separated by function: RS485 is used for the structural bus, while RS232 is used for the environmental node.

The BridgeWatch−Raspberry communication uses an ad-hoc software protocol, GeoProtocol. Each packet includes a packet counter, sender and receiver identifiers, a command byte, data length, payload and bytes for cyclic redundancy checks. The receiver returns an acknowledgement message when a packet is correctly interpreted, enabling detection of communication errors and retransmission if required. This structure was selected because unattended SHM operation requires robust data integrity checks over long cable runs.

**Figure 6:**
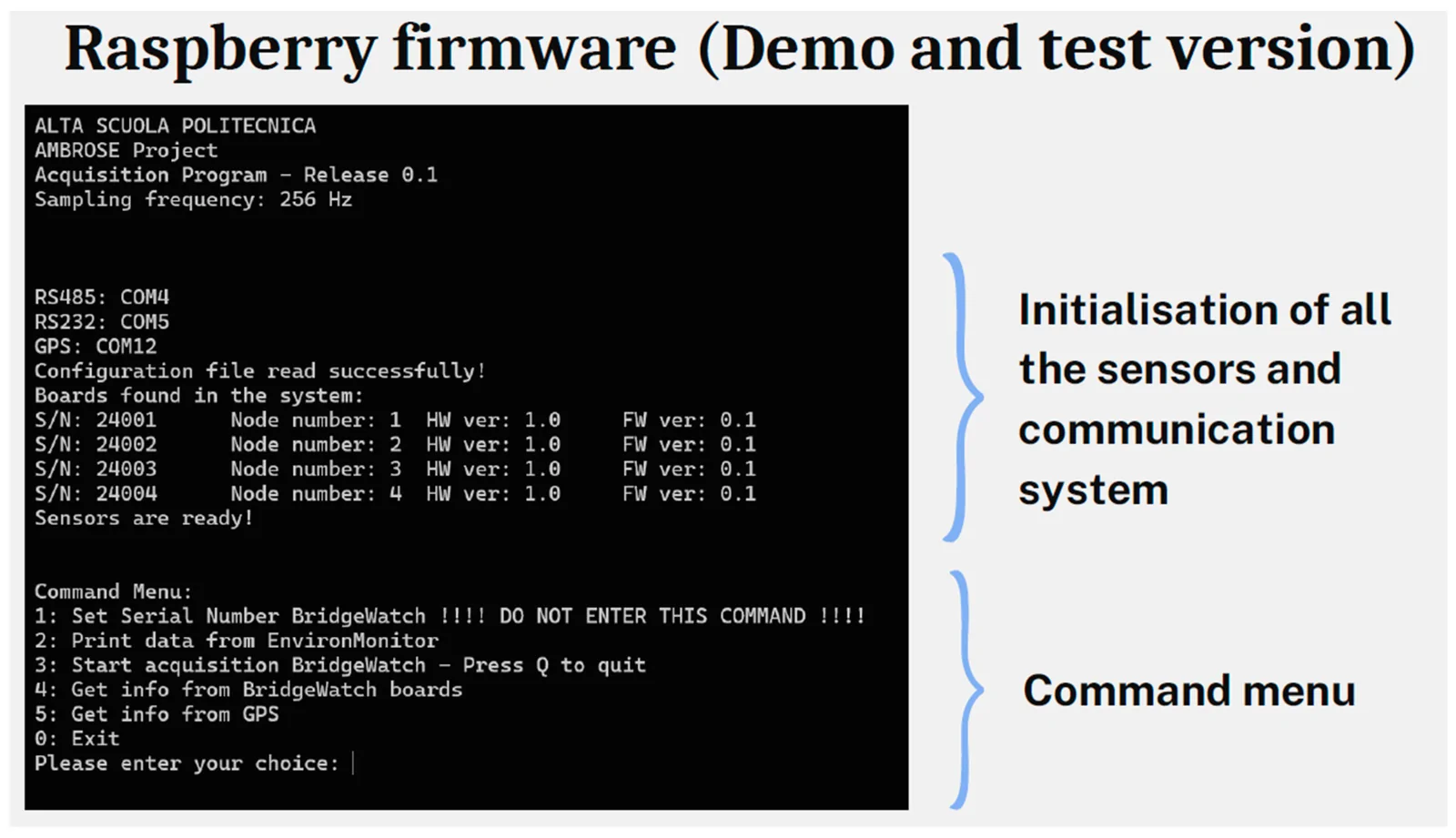
Raspberry Pi firmware interface for the laboratory and demo version, showing sensor initialisation, board detection and the command menu.

The EnvironMonitor−Raspberry communication uses an NMEA 0183-style sentence format over RS232. This choice simplifies parsing because similar formatting is also used by GPS devices. The Raspberry Pi firmware manages initialisation, board detection, serial number configuration, environmental data display, structural data acquisition, debug information and GPS information. Figure 6 shows the demo and laboratory interface with the initialisation output and command menu.

The firmware logic is menu-driven during laboratory use, while the acquisition function writes synchronised data to files, according to the Raspberry Pi control routine reported in Supplementary Materials (Section B). During acquisition, the program writes the structural and environmental files with coordinated timestamps.

At the node level, the ADXL355 accelerometer is sampled at 256 Hz using the rising edge of a PWM signal generated by each STM32 microcontroller’s timer. The acquisition is organised into 60 s cycles: 59 s is devoted to data acquisition. The remaining second is used to transfer and write the acquired data and to re-synchronise the BridgeWatch nodes. At the beginning of each new acquisition cycle, the Raspberry Pi sends the E_START command in broadcast mode, causing the sampling timers of all BridgeWatch nodes to stop and restart from the same initial condition. This periodic reinitialisation limits the accumulation of relative timing drift among the independent local clocks. The system does not employ a continuously distributed hardware sampling clock; instead, the local time bases are periodically realigned through the broadcast command.

To improve the stability of the local sampling time base, each BridgeWatch microcontroller is equipped with an external oscillator rather than relying exclusively on the less accurate internal microcontroller clock. The external time base drives the timer that generates the 256 Hz PWM sampling trigger. Nevertheless, the different nodes do not share a continuously distributed hardware sampling clock; consequently, a residual relative clock drift may still develop during each 59 s acquisition interval. The periodic broadcast reinitialisation is intended to prevent this relative drift from accumulating over successive acquisition cycles.

It should be noted that, in the implementation validated in this study, the Raspberry Pi performs system initialisation, node identification, command transmission, data reception and parsing, synchronisation management, timestamp assignment and local file generation. FFT and modal-identification analyses, as presented in the following Section 6, were conducted offline in MATLAB R2025b on the laboratory computer after acquisition. For the intended field implementation, the same Raspberry Pi can also serve as an edge-computing unit.

On the EnvironMonitor microcontroller, environmental data are updated by a combination of periodic sampling and interrupt handling. Rainfall is obtained from the elapsed time between tipping-spoon pulses, while a timer clears the rain-rate value if no new tick occurs within a prescribed interval. Wind speed is derived from the number of anemometer pulses over a one-second sample period using the manufacturer’s relationship and then converted to SI units. Water level and wind direction are obtained by ADC conversion after conditioning. In the current work, which focused on hardware design, these measurements were collected and stored separately. However, future research will attempt to combine these measurements to produce one or more low-dimensional, environment-informed damage metrics.

## 6. Laboratory Experimental Validation

A laboratory validation campaign was designed to assess the proposed monitoring system through a sequence of controlled tests. The experimental program included verification of communication and synchronisation, frequency-response characterisation using a laboratory shaker, modal identification on a benchmark scaled building, evaluation of accelerometer sensitivity to temperature variations, and validation of the environmental sensing module under both real and simulated operating conditions, as detailed in the following subsections.

### 6.1. Laboratory Test Architecture

**Figure 7:**
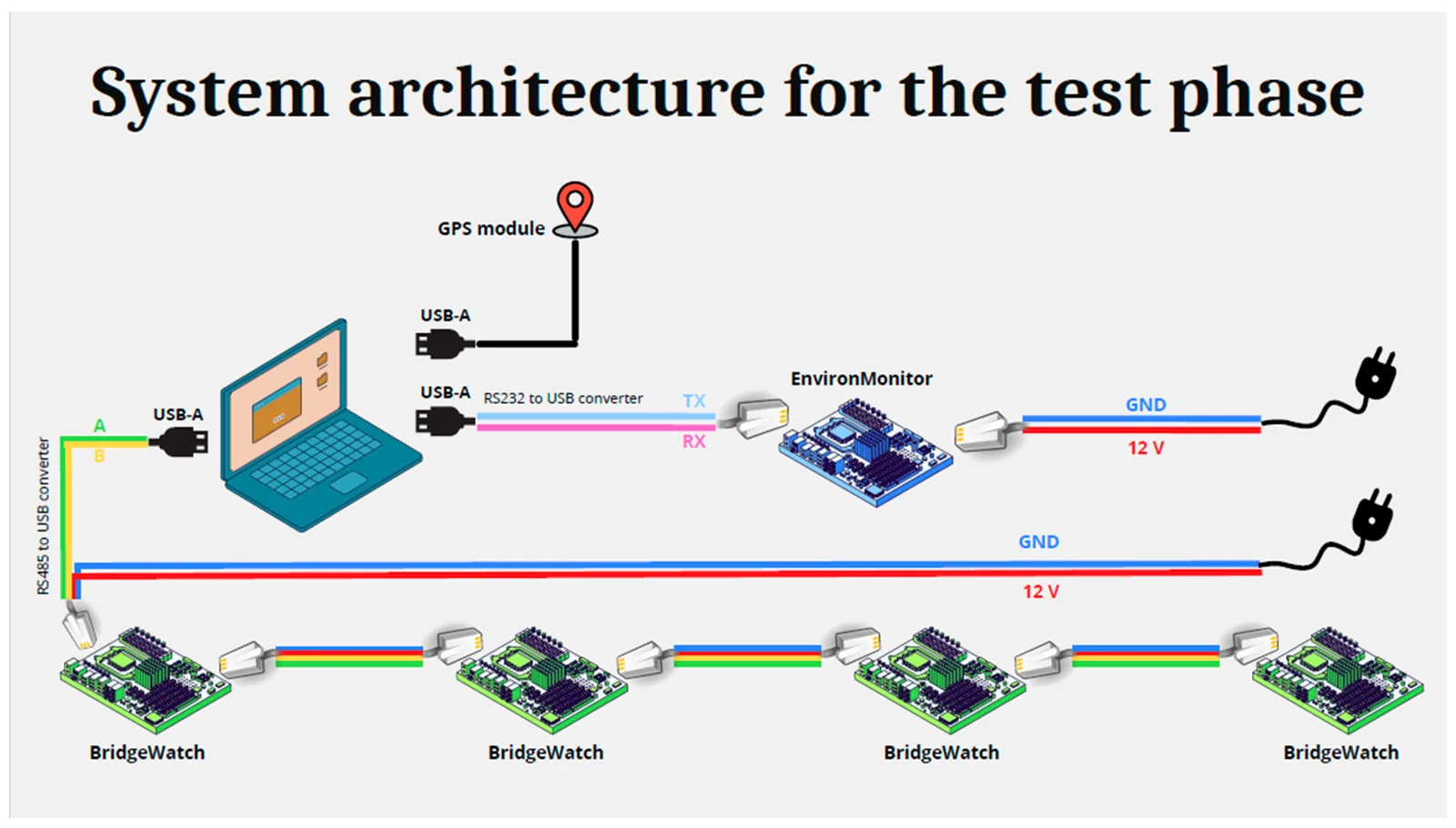
Architecture adopted during the laboratory test phase, using a laptop as the acquisition coordinator and direct laboratory power supplies.

For the laboratory campaign, the field architecture was simplified to reduce setup time. The battery and solar supply were not used, and a laptop replaced the Raspberry Pi as the central acquisition unit. Figure 7 shows the test-phase architecture. This configuration preserves the communication topology and board-level behaviour while making access to serial ports, power supplies and data files more convenient.

**Figure 8:**
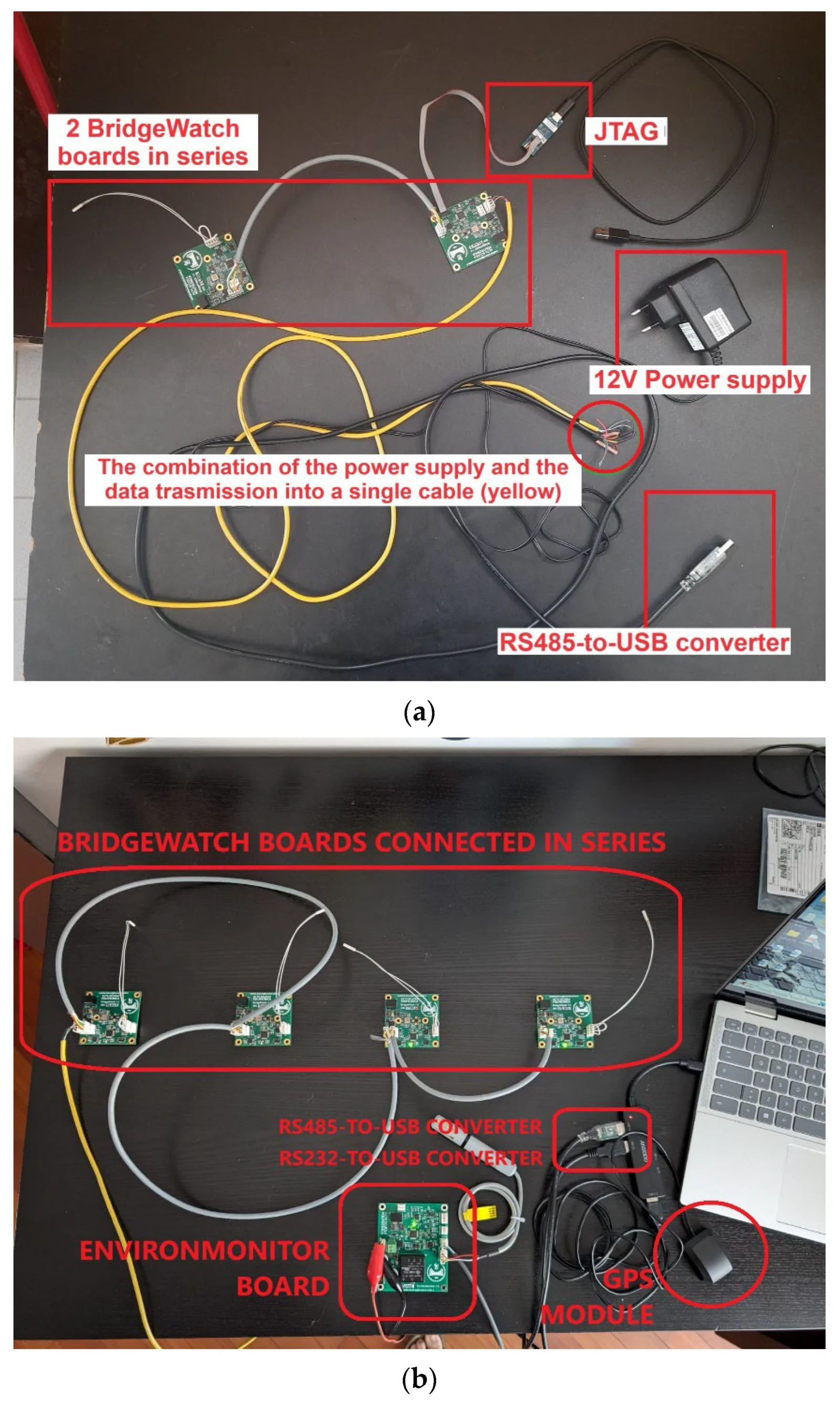
(**a**) Laboratory configuration with two BridgeWatch boards connected in series, the shared data-power cable and the RS485-to-USB converter. (**b**) Complete laboratory test configuration with four BridgeWatch boards in series, EnvironMonitor, RS485 and RS232 converters, and the GPS module.

Figure 8 shows two representative laboratory configurations. Figure 8a illustrates the BridgeWatch serial connection with two boards in series, the 12 V supply, the combined data-power cable, JTAG access and RS485-to-USB conversion. Figure 8b shows a complete configuration with four BridgeWatch boards, one EnvironMonitor board, one GPS module, and the RS485 and RS232 converters connected to the laptop. The latter corresponds to the exact configuration used for the preliminary BridgeWatch communication and synchronisation test described in the following subsection.

### 6.2. Preliminary BridgeWatch Communication and Synchronisation Test

**Figure 9:**
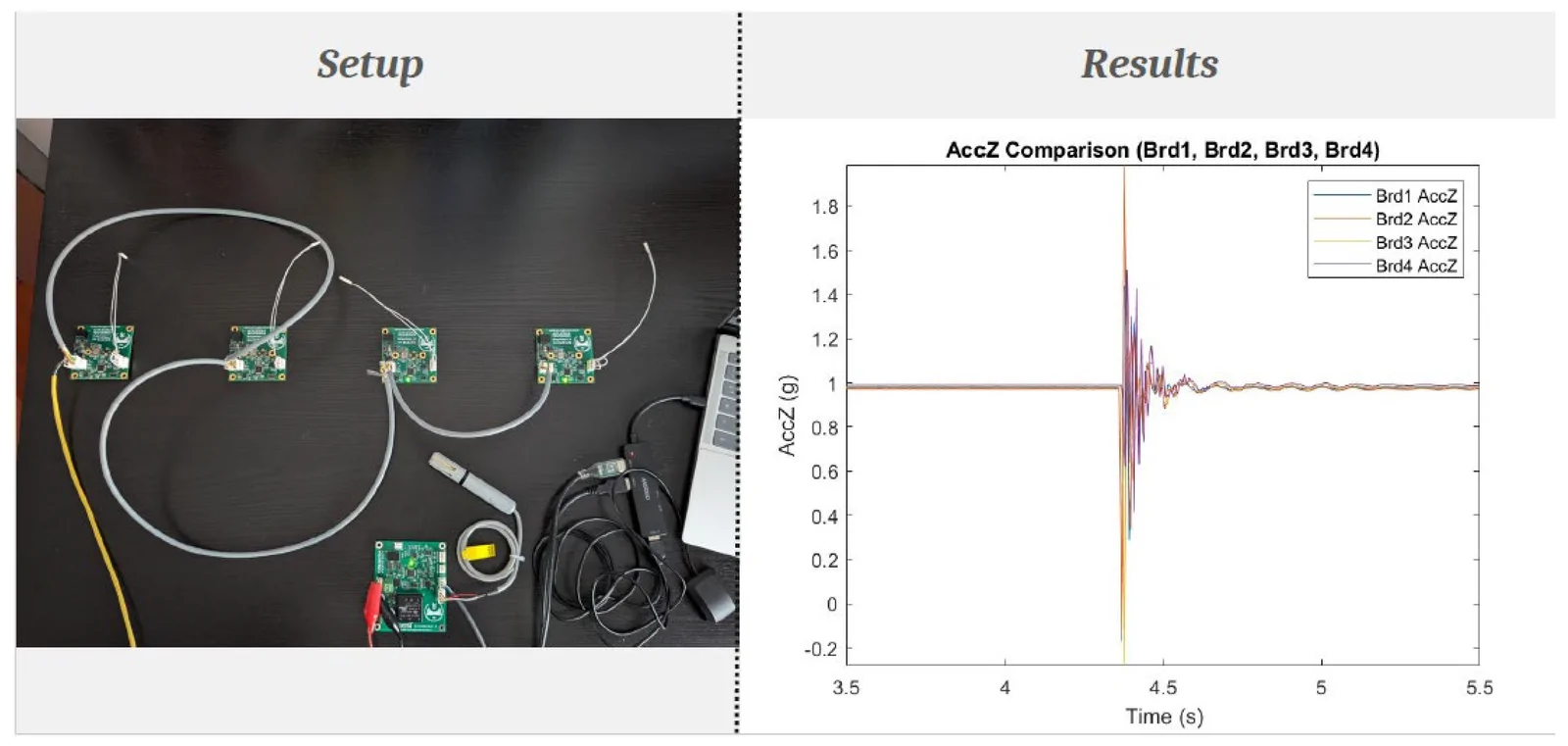
Preliminary accelerometer test: four BridgeWatch nodes connected in series (**left**) and the corresponding *z*-axis acceleration response after an impulsive force. Only the time series corresponding to the four vertical accelerometer channels are shown (**right**).

The preliminary test was designed to verify initialisation, sensor acquisition, RS485 communication and data logging before moving to calibrated dynamic tests. Four BridgeWatch boards were connected in series, as described above, and placed on a table. After starting the acquisition, the table was hit with a single impulse, and the *z*-axis acceleration was plotted for all boards. Figure 9 shows the setup and the resulting acceleration response.

The impact is evident as a sharp peak in all four *z*-axis acceleration signals, providing an initial qualitative verification that the acquisition and communication chains were operating correctly. Relative signal alignment was subsequently assessed through normalised cross-correlation. One BridgeWatch channel was selected as the reference, and the lag corresponding to the maximum absolute cross-correlation was determined for each of the other channels. The relative delay was calculated as $\tau_{ij}=k_{ij}/f_{s}$, where $k_{ij}$ is the estimated lag in samples and $f_{s}$ = 256 Hz is the sampling frequency. Accordingly, one sample corresponds to 3.906 ms. This value represents the temporal resolution of an integer-sample delay estimate and should not be interpreted as the measured delay between nodes. The cross-correlation maxima occurred at zero lag for all board pairs with the reference channel. Therefore, no inter-board delay was resolvable at the acquisition time resolution of 3.906 ms. The zero-lag result demonstrates sample-level alignment over the analysed impulse record but does not constitute a sub-sample metrological characterisation of synchronisation. Because the BridgeWatch nodes use independent local clocks between successive re-synchronisations, a residual relative drift may develop within each 59 s acquisition interval, although the periodic E_START procedure prevents such drift from accumulating indefinitely over consecutive acquisition cycles. At the adopted sampling frequency, one sample corresponds to 3.906 ms. A one-sample temporal offset would correspond to a phase shift of approximately 7.0° at 5 Hz, 14.1° at 10 Hz and 28.1° at 20 Hz. This offset does not alter the spectral-peak frequency identified independently for each channel. The available impulse experiment was not designed as a multi-cycle metrological characterisation of clock skew; therefore, it does not provide a measured maximum pre-resynchronisation drift or a sub-sample timing mismatch. Dedicated characterisation of inter-node skew immediately before successive re-synchronisation events will form part of the forthcoming in situ full-scale bridge validation campaign. Accordingly, no indications can be reported, at this stage, regarding long-duration clock-drift performance.

### 6.3. Shaker Test

The shaker test was the key experimental validation used to verify the frequency response of the BridgeWatch board under a controlled sinusoidal excitation. In this regard, it was designed primarily as a controlled functionality check of BridgeWatch frequency acquisition. A single BridgeWatch node was fixed to a laboratory shaker, and sinusoidal commands were imposed at prescribed amplitudes and frequencies.

Each acquisition contained 15,104 samples collected during 59 s at 256 Hz. The corresponding FFT-bin spacing is therefore $\Delta f=f_{s}/N=0.01695$ Hz. The acquired acceleration time series were processed offline in MATLAB, using detrending, a Hanning window to reduce spectral leakage in frequency-domain analysis, and FFT-based peak extraction. Furthermore, to assess the amplitude response of the complete measurement chain, the amplitude of the fundamental sinusoidal component was also estimated from each acceleration record. After the removal of the mean value, the records were divided into non-overlapping 4 s windows, and a least-squares sinusoidal fit was performed by optimising the excitation frequency around the dominant FFT peak. The reported BridgeWatch amplitude corresponds to the mean peak amplitude obtained over the analysed windows. This approach was preferred over the direct use of the maximum FFT bin because the latter could be affected by window-coherent gain and spectral leakage.

**Figure 10:**
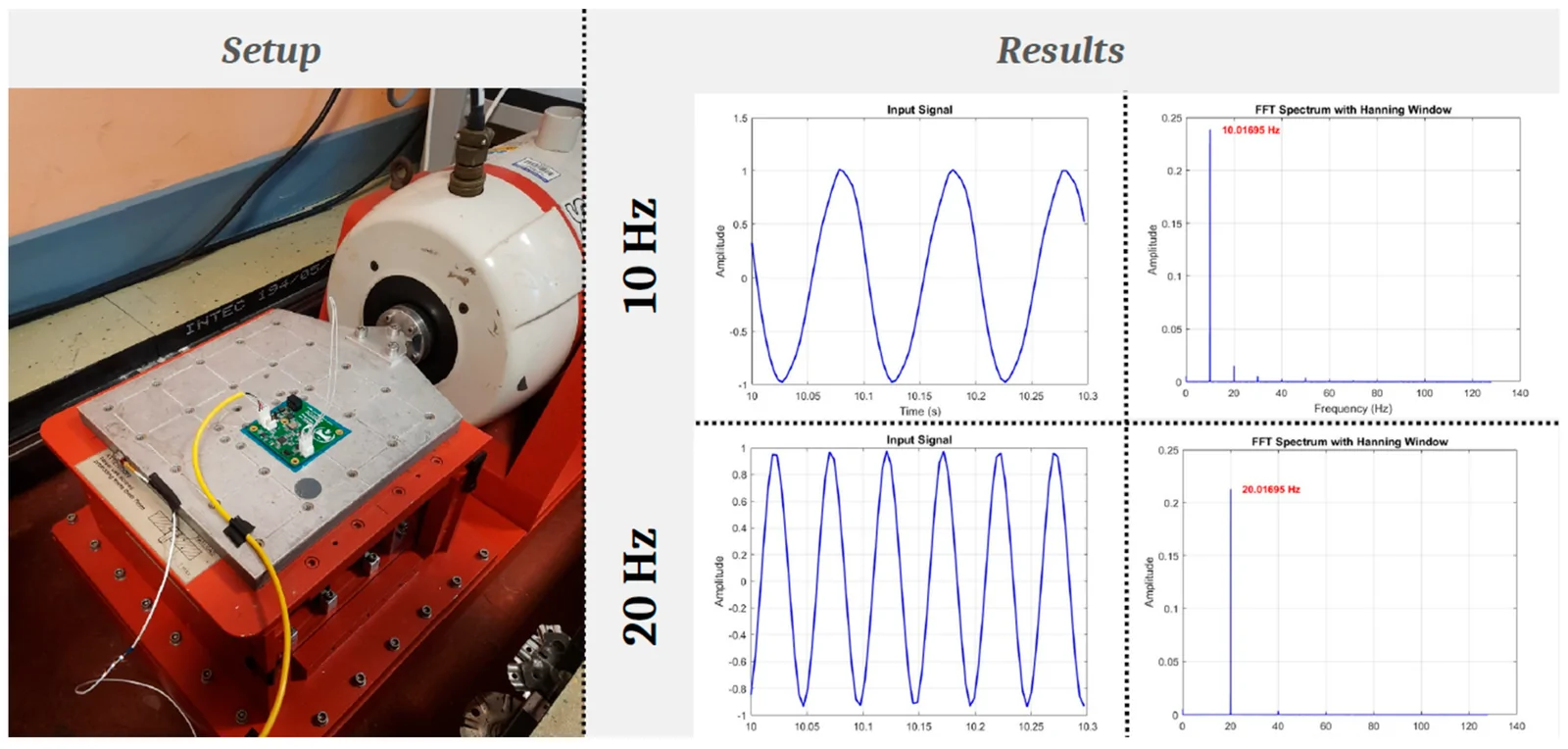
Shaker validation: laboratory setup with one BridgeWatch board mounted on the shaker (**left**), and representative 10 Hz and 20 Hz acquisitions with their FFT spectra (**right**). The spectra are used here to verify recovery of the commanded frequency; amplitude values are compared only with nominal shaker setpoints.

Five tests were performed, encompassing different input driving frequencies, in the same order used in Table 1: (i) 5 Hz at 0.5 g, (ii) 10 Hz at 1 g, (iii) 20 Hz at 1 g, (iv) 50 Hz at 1 g and (v) 100 Hz at 1 g. The 5 Hz command amplitude was reduced because maintaining constant acceleration at low frequency requires a larger shaker displacement. Representative 10 Hz and 20 Hz results (cases ii and iii) are shown in Figure 10; the complete set is summarised, as said, in Table 1.

The extracted peak frequencies match the imposed frequencies with a signed percentage deviation (${diff}_{f}=100\cdot (f_{identified}-f_{nominal})/f_{nominal}$) below 0.2% for all non-ideal cases and exactly 5.00 Hz for the 5 Hz case. The 100 Hz test is close to the Nyquist frequency at the 256 Hz sampling rate, so the time-domain waveform is reconstructed with slightly reduced accuracy, but the FFT still correctly identifies the imposed frequency. Note that this assessment quantifies consistency with the nominal shaker command and should not be interpreted as an independently calibrated absolute frequency accuracy of the node.

Similarly, the tabulated signed relative amplitude difference (${diff}_{A}=100\cdot (A_{identified}-A_{nominal})/A_{nominal}$) provides an assessment complementary to that of frequency identification. The measured fundamental amplitudes correspond to deviations not exceeding approximately 5% from the nominal shaker settings, except for the last two cases with the highest set input frequencies (50 Hz and 100 Hz). This observed attenuation increases with frequency and may be attributed to frequency-dependent phenomena such as the shaker/table transfer response, mounting and the sensor/acquisition filtering chain. The completed experiment does not allow retrospective separation of these effects because no calibrated, co-located reference accelerometer was mounted on the shaker.

### 6.4. Scaled Building Model Test

The model-building test was performed in the laboratories of the Department of Structural, Geotechnical and Building Engineering (DISEG) at Politecnico di Torino, using a three-storey aluminium frame. The structure, which was also used for the tests reported in Dragonetti et al. [41], consists of four rectangular-section columns, three square plates with 400 mm sides and 300 mm inter-storey spacing, steel angle connections and a 500 mm × 500 mm steel base resting on four supports. A more detailed discussion of the mock building can be found in [41]. The purpose of this test was to verify whether BridgeWatch data could be used to identify the natural frequencies of a known benchmark structure, by comparing them with the identifications from those previous tests [41]. In addition, the test assessed whether the measured response was consistent with that recorded by reference accelerometers installed on the same structure. These reference sensors are wireless devices developed by the Moni2BSafe research team led by Prof. Abruzzese at the University of Rome Tor Vergata (https://moni2bsafe.com/en/home-english/, last accessed on 12 September 2026). This system includes an ADXL355 MEMS accelerometer, the same sensor used in the BridgeWatch boards. It has recently been employed in field tests of the Santa Caterina Church [42] and other significant structures. For all these reasons, although it represents a multi-storey shear-type building rather than a bridge, this scaled-down case study was considered the best available option. Notably, since the Moni2BSafe reference chain uses the same ADXL355 sensing element, it does not constitute an independent sensor-level calibration reference; it is rather intended as a separate acquisition-chain consistency check under the same structural response.

The benchmark structure was selected because independently identified modal parameters and a separate reference acquisition system were available, thereby providing known quantities against which the complete BridgeWatch measurement chain could be assessed. The purpose of this experiment was, consequently, to validate multi-channel acquisition, natural-frequency extraction and modal-analysis capabilities under controlled conditions. The three-storey frame is not intended to reproduce the dynamic behaviour of a full-scale bridge. In particular, bridge-specific aspects, such as lower-amplitude operational vibrations under traffic excitation, different damping levels, installation conditions and long-term environmental variability, remain to be assessed in the forthcoming full-scale field campaigns.

**Figure 11:**
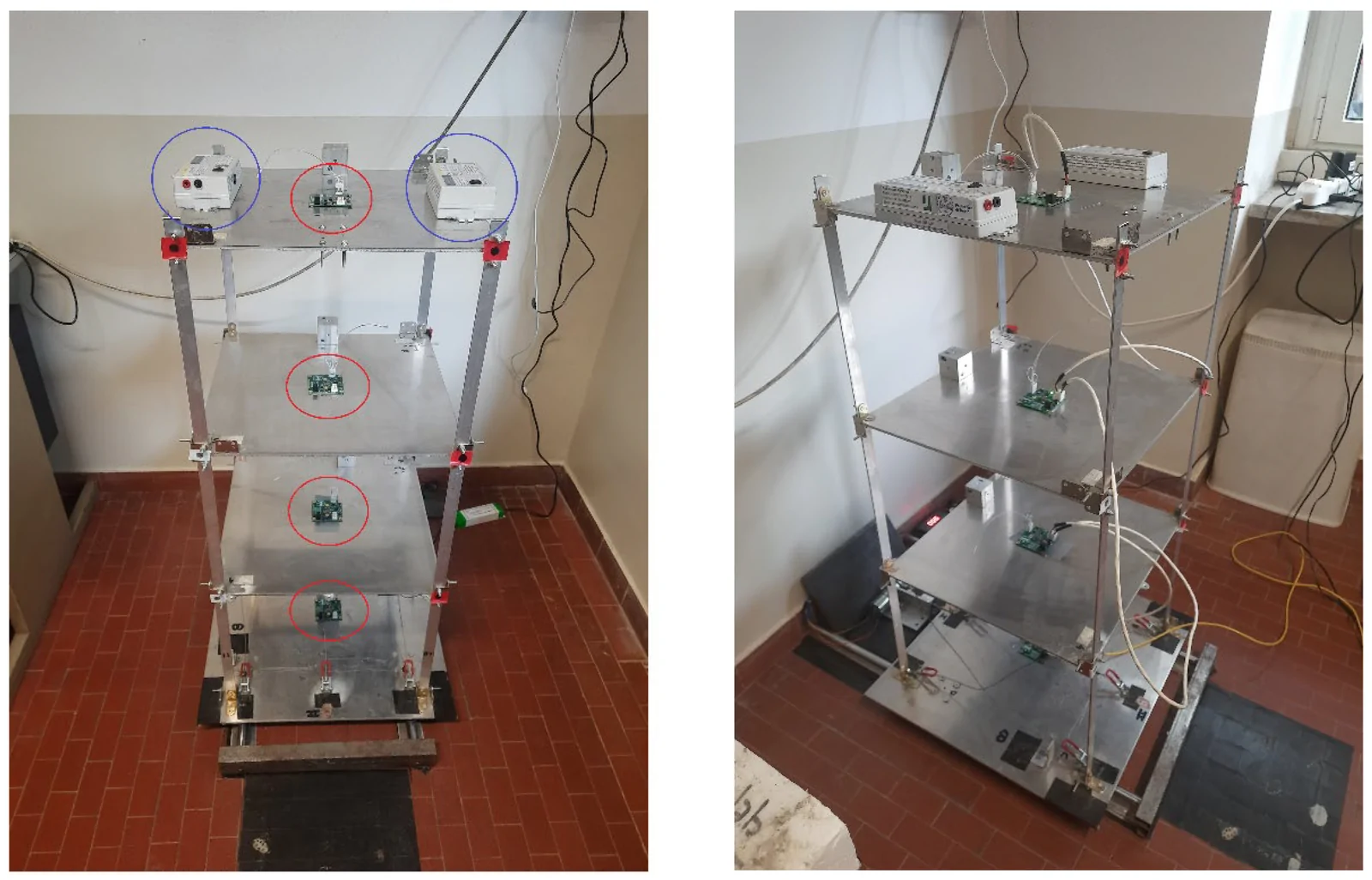
The single-bay, three-storey laboratory building model with BridgeWatch nodes (highlighted in red) and reference accelerometers (highlighted in blue) installed at different levels, as seen in the front view (**left**) and in the side view (**right**).

**Figure 12:**
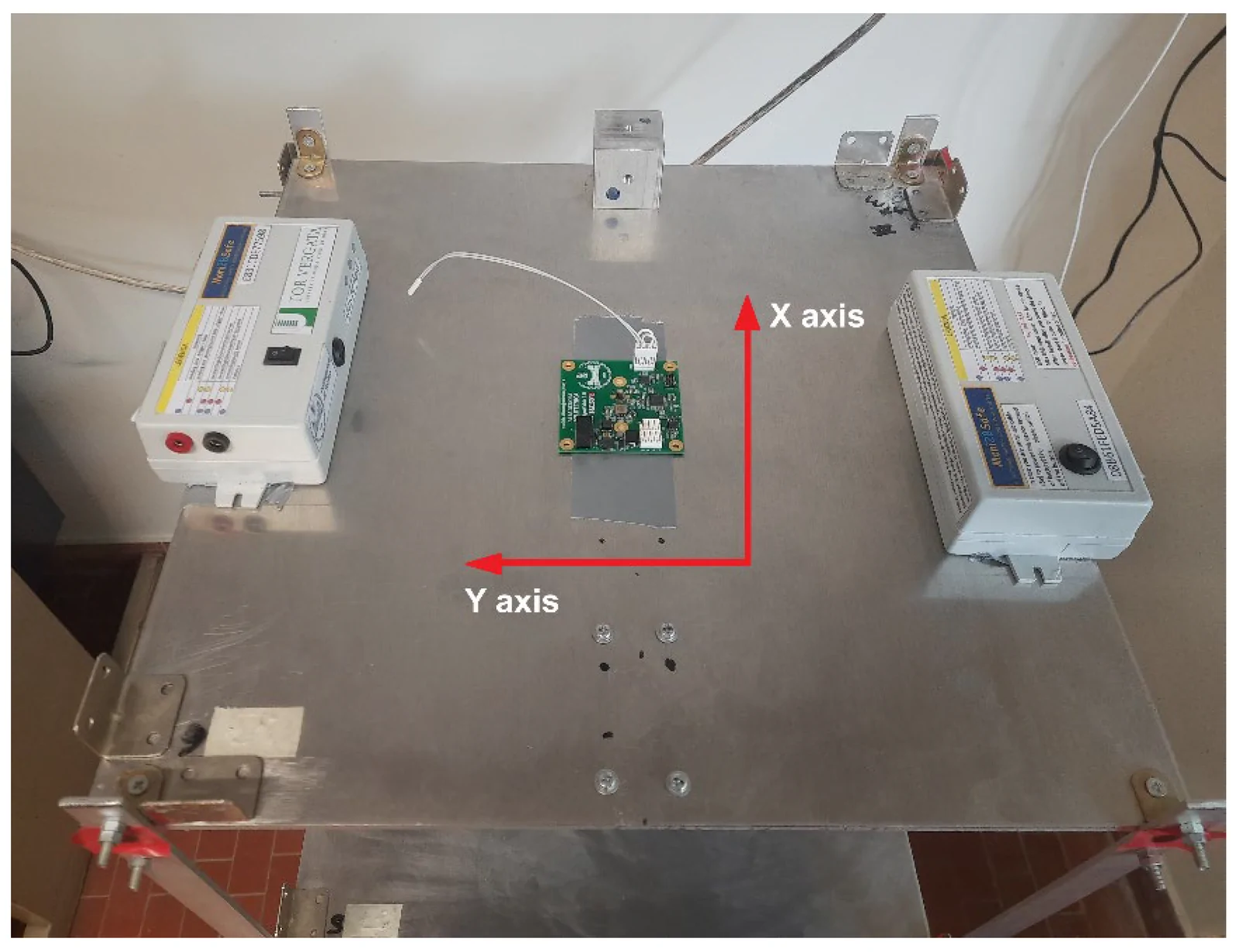
Top-plate setup and reference frame for the model-building validation test.

Figure 11 shows the sensor layout on the model, and Figure 12 portrays the top plate with the reference axes. BridgeWatch nodes were distributed at different levels of the structure, while reference accelerometers were placed on the top plate. The test included both ambient vibration and forced vibration tests. Ambient data were analysed by FFT and Data-Driven Stochastic Subspace Identification (SSI-DATA). Forced response was obtained by hammer hits applied horizontally to the top plate in the x- and y-directions, with a sufficient pause between blows to allow free decay, following the same experimental setups described in Dragonetti et al. [41].

**Figure 13:**
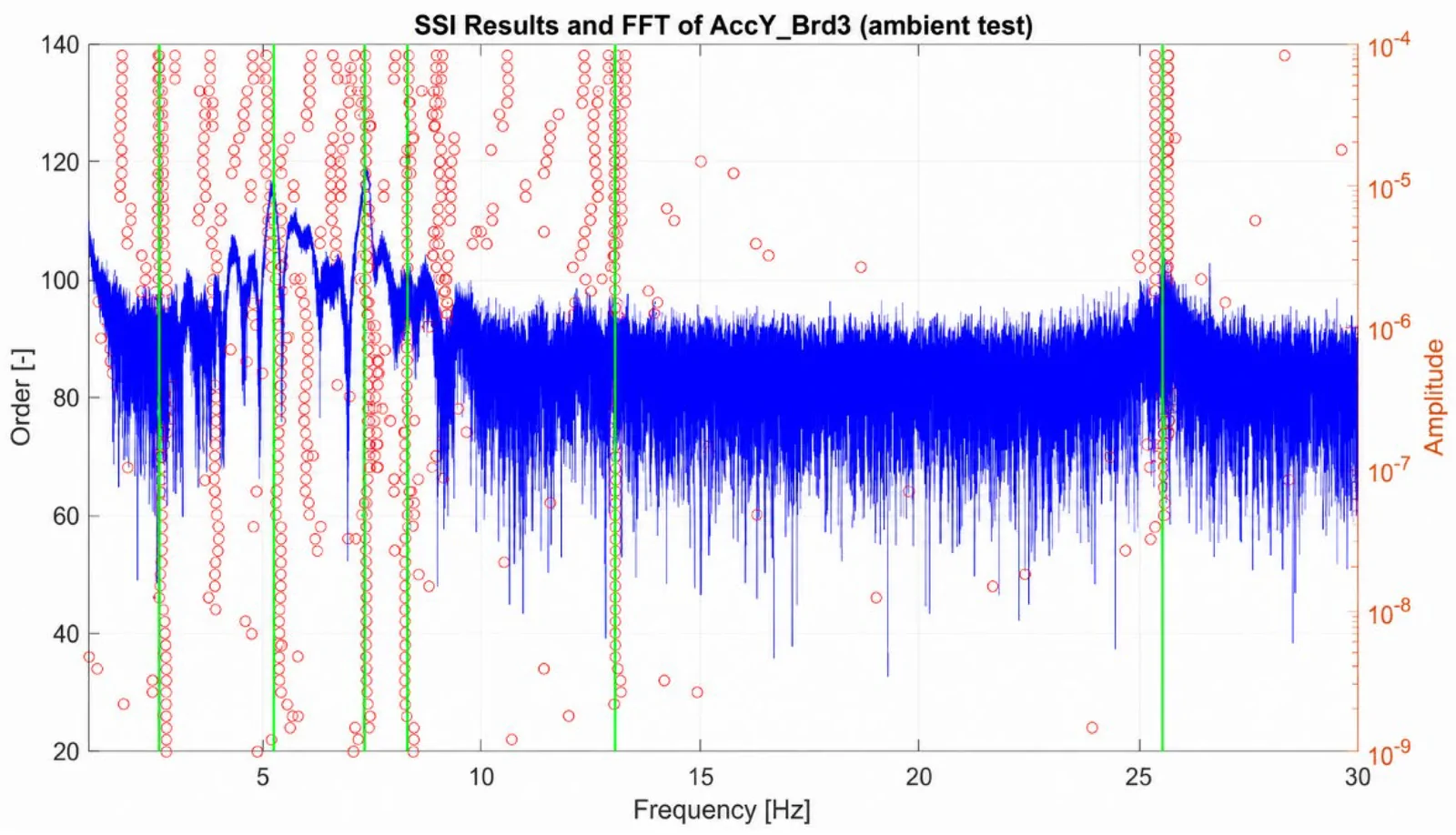
Representative ambient vibration test-derived modal identification result on the three-storey benchmark: SSI-DATA stabilisation diagram and corresponding spectral information used to identify stable modal frequencies.

Figure 13 graphically reports representative results for the extraction of natural frequencies. The stabilisation diagram from SSI-DATA and the FFT spectrum identify stable poles and spectral peaks. The first six identified natural frequencies are reported in Table 2 and compared with reference values obtained in previous experimental work on the same benchmark. The measured frequencies are systematically lower than the values reported in Dragonetti et al. [41]; however, this was expected, as it is consistent with the additional mass of the installed sensors and reference accelerometers (with respect to the corresponding unloaded configuration investigated there), as well as with the possible flexibility and damping introduced by adhesive mounting. Conversely, the comparison with the reference sensors (Moni2BSafe) is optimal, never diverging by more than 0.06 Hz in the target modes (the first six) except for mode 4; this should also be interpreted considering the potential effects of the Moni2BSafe sensor enclosure, which the Authors were not allowed to remove from these tests. Quantitatively, the BridgeWatch estimates are approximately 3.5–12.1% lower than the earlier unloaded values reported by Dragonetti et al. [41] reported values, while the comparison with Moni2BSafe differs by no more than 0.06 Hz for five modes and by 0.29 Hz (approximately 3.6%) for mode 4. The former difference is plausibly influenced by the changed mass/mounting configuration due to the sensors’ presence, whereas the latter indicates that even the same-sensor comparison should not be interpreted as an absolute calibration.

As a further analysis, an approximate calculation of the signal-to-noise ratio of the two acquisition systems was made. The SNR was measured as the ratio of the peak height to the root-mean-square deviation (RMSD) value of the noise floor.

**Figure 14:**
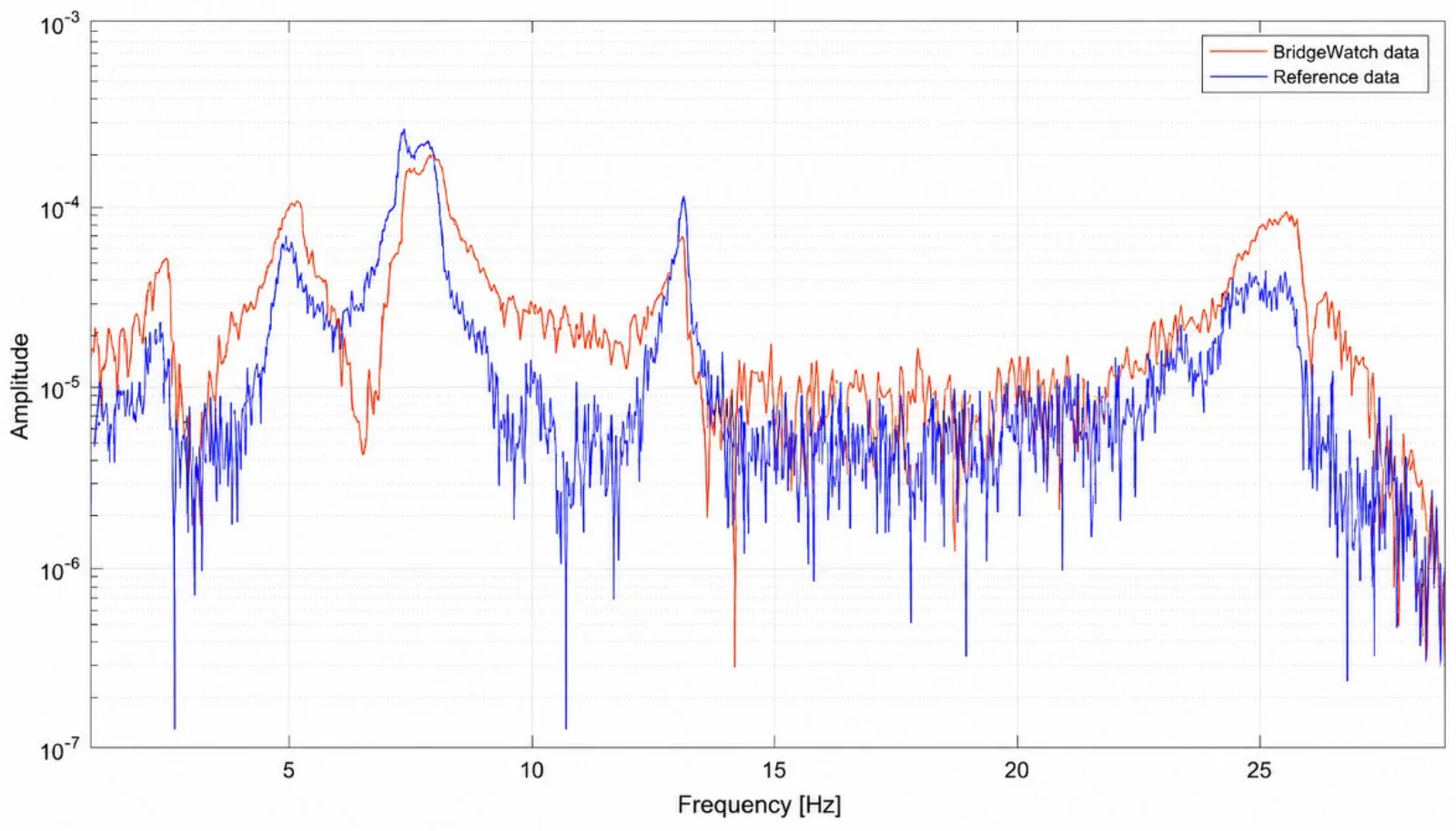
Frequency-domain comparison for one hammer-impact record on the model building: BridgeWatch spectrum (red) and the separate Moni2BSafe acquisition-chain spectrum (blue).

Noise was evaluated in the 14–20 Hz band, and the third-mode peak was used as the signal level. The resulting approximate SNR values are SNR = 26.21 dB for the Moni2BSafe reference chain and SNR = 26.63 dB for BridgeWatch. This close agreement indicates that the proposed low-cost node can capture the benchmark’s primary modal information with signal quality comparable to, or slightly better than, the reference acquisition setup (see Figure 14 for a visual comparison). However, because both systems use the ADXL355 sensing element, this comparison does not establish an independent assembled-node noise floor or absolute sensor accuracy; rather, it shows consistency in the complete BridgeWatch acquisition chain under the same structural excitation.

### 6.5. Climatic-Cell Test

**Figure 15:**
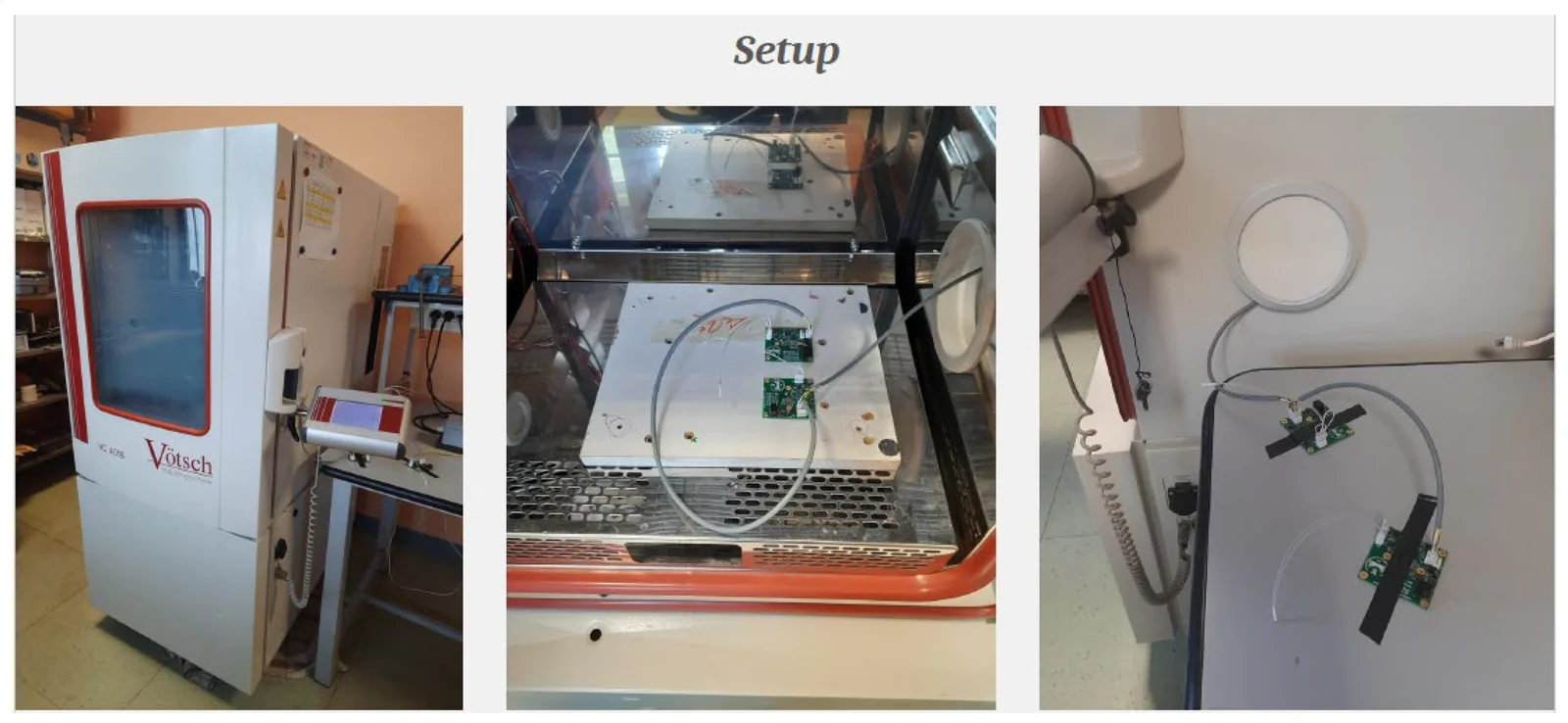
Climatic-cell setup for evaluating temperature sensitivity of BridgeWatch acceleration readings. Brd1 and Brd3 remained outside at room conditions, whereas Brd2 and Brd4 were placed inside the cell and subjected to the programmed thermal sequence.

The climatic-cell test evaluated the sensitivity of BridgeWatch accelerometer readings to temperature variations. Two BridgeWatch boards (Brd2 and Brd4) were placed inside the climatic cell, while two (Brd1 and Brd3) were kept at room temperature. The cell was programmed to cycle through temperatures from room conditions to +20 °C, +5 °C, −10 °C, +20 °C, +40 °C, +60 °C and back to the initial value (+20 °C). A constant relative humidity of 25% was maintained throughout the whole test. Figure 15 shows this test setup, including the climatic cell and the board arrangement.

**Figure 16:**
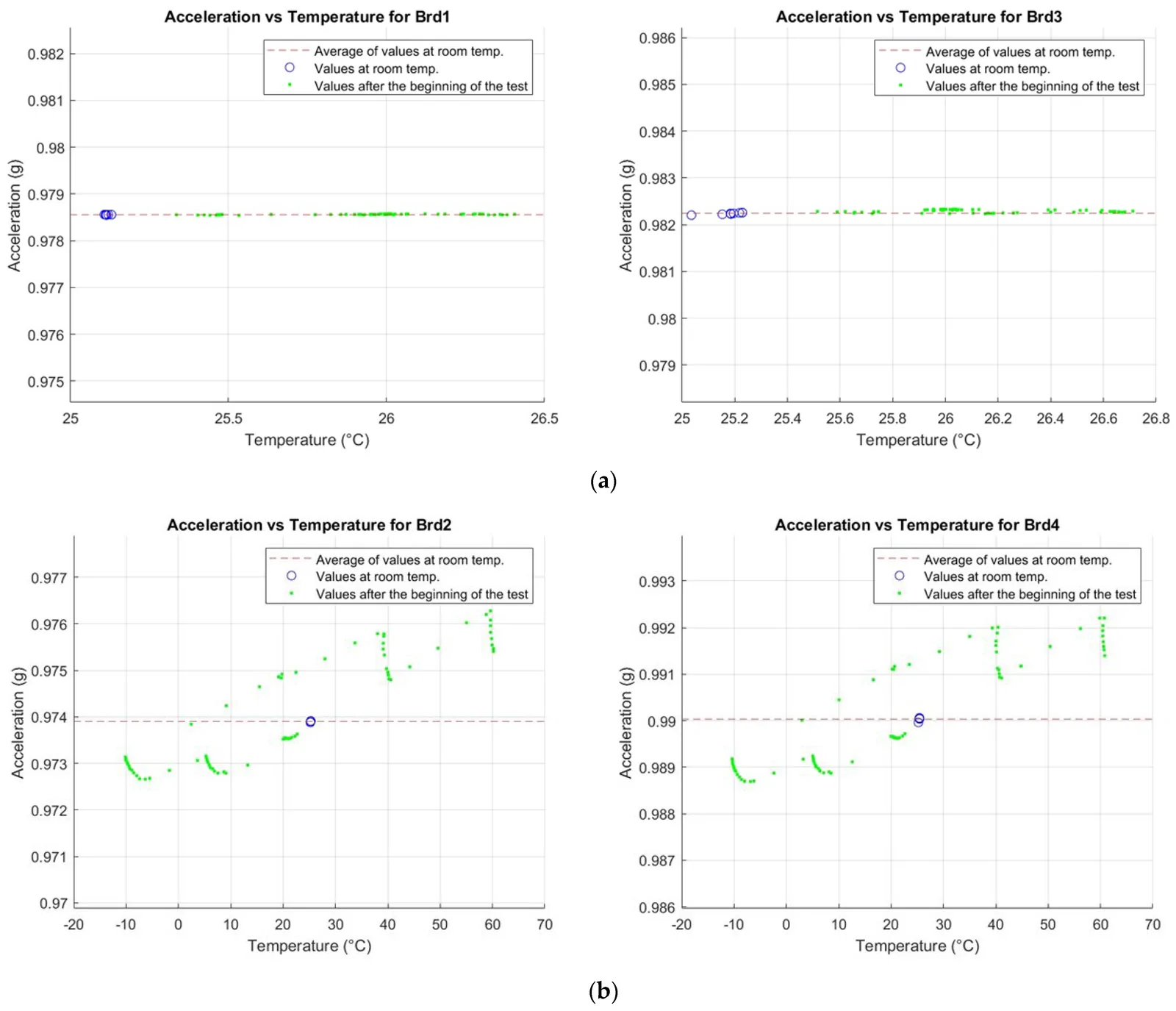
Climatic-cell results: (**a**) Brd1 and Brd3, maintained outside the cell, showing near-constant *z*-axis acceleration under room-temperature reference conditions; (**b**) Brd2 and Brd4, placed inside the cell, showing the temperature-dependent *z*-axis acceleration trend over the imposed thermal sequence.

The data were processed using a dedicated MATLAB code. Figure 16a,b present the full results. As mentioned, this experiment aimed to characterise acceleration-offset sensitivity to temperature; tilt accuracy was not tested.

As shown, the room-temperature board exhibits nearly constant acceleration around the baseline. Conversely, the board exposed to the thermal sequence shows a slight temperature-dependent trend: the measured *z*-axis acceleration is slightly lower at low temperature and increases as temperature rises. This behaviour is consistent with a modest thermal sensitivity in the accelerometer response. However, in practical terms, the thermal effects are consistent with commercial products and negligible in real-life field applications (less than 5 mg over the [−10, +60] °C range).

### 6.6. EnvironMonitor Environmental Sensor Test

**Figure 17:**
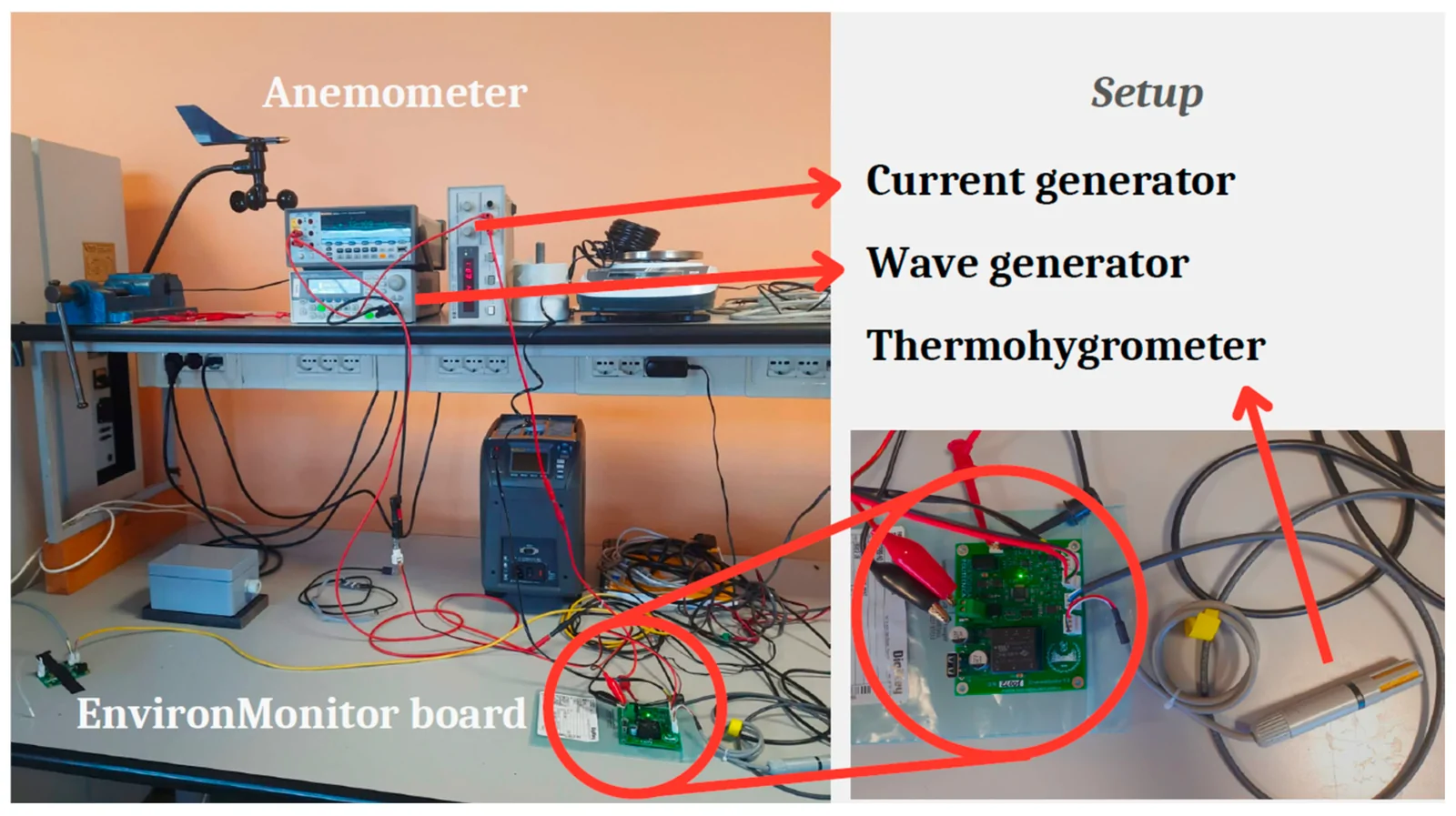
EnvironMonitor signal-conditioning validation setup, including the physical anemometer and thermohygrometer together with the current generator used to emulate the 4–20 mA water-level output and the waveform generator used to emulate rain-gauge tipping pulses.

The EnvironMonitor validation verified sensor interfacing and environmental data transmission. While the anemometer and thermohygrometer were tested using their physical inputs, the water-level sensor and rain-gauge outputs were simulated electrically, as the available facilities and tools could not physically reproduce varying rain intensity and river water levels under controlled conditions. In addition, the main objective of this testing stage was to validate the EnvironMonitor acquisition chain from the standardised sensor electrical output to the final engineering quantity, rather than to revalidate the physical sensing principle of the commercial transducers. The water-level sensor’s current output, expected to range from 4 mA to 20 mA for a 0–30 m range, was reproduced using a current generator. Reproducing the current output directly exercises the input protection and conditioning circuitry, current-to-voltage conversion, buffer stage, ADC, firmware scaling, serial transmission and final data parsing. The rain-gauge tipping-spoon signal, corresponding to 0.2 mm per tip, was reproduced using a square wave between 0 V and 3.3 V. The anemometer and thermohygrometer were connected to their actual inputs. Figure 17 shows the general test setup.

A component-level estimate also indicates the resolution and the principal electronic uncertainty contributions of the water-level acquisition channel. The 4–20 mA signal was converted through a 120 Ω, 0.1% shunt resistor and digitised by the 12-bit microcontroller ADC using a nominal 2.5 V reference. The ADC quantisation step was therefore approximately 0.610 mV/count, equivalent to 5.09 µA/count and approximately 9.54 mm/count over a 0–30 m level range mapped onto 4–20 mA. The 0.1% shunt tolerance corresponded to an approximately 30 mm full-scale gain contribution, while the 1% initial tolerance of the voltage reference would correspond to an approximately 0.30 m full scale if left uncalibrated. Its 50 ppm/°C temperature coefficient corresponded to approximately 1.5 mm/°C over the 30 m span. These values are conservative component-level bounds rather than a complete uncertainty budget for the assembled system. Calibration can further reduce fixed-gain and offset contributions.

**Figure 18:**
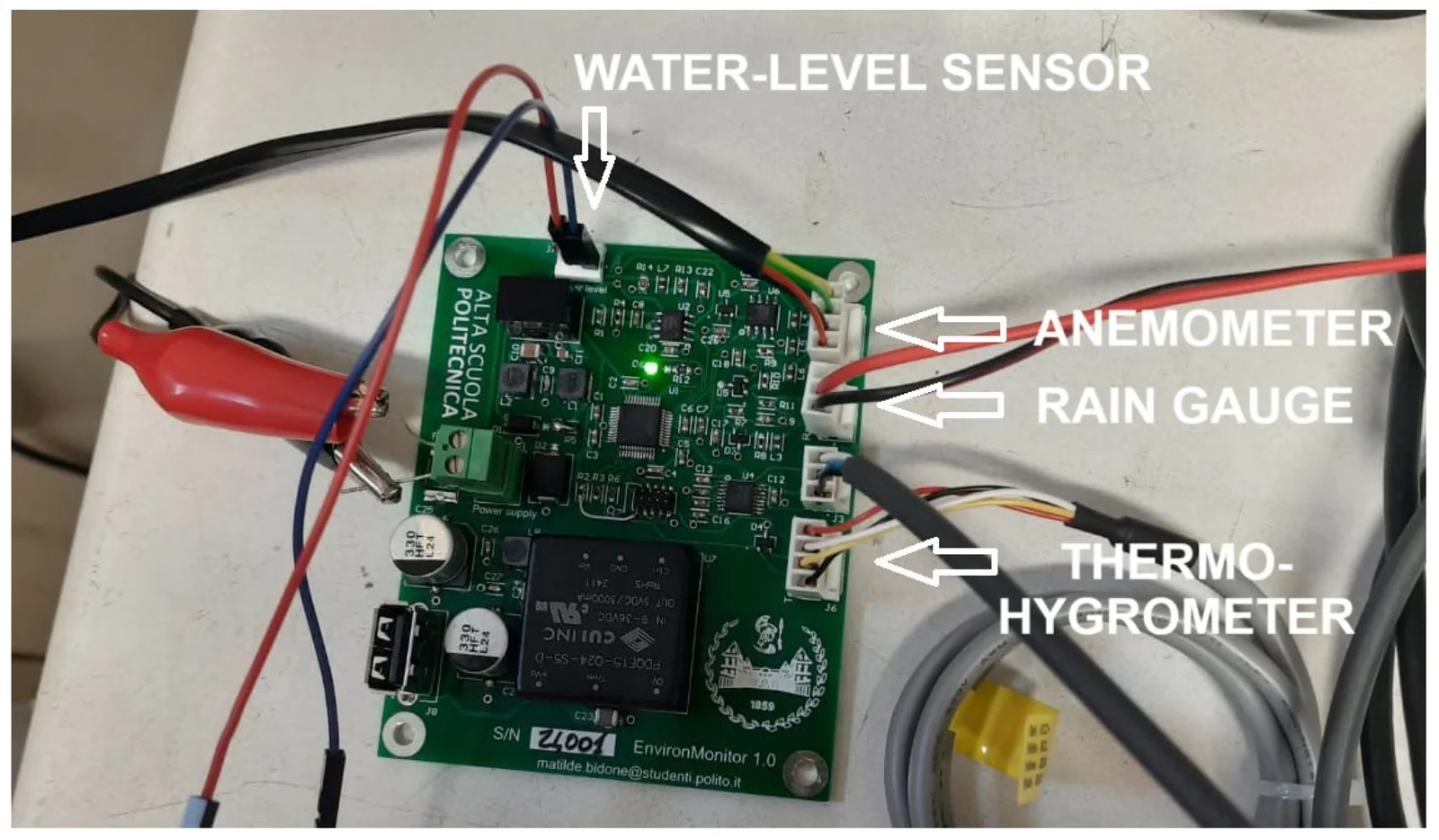
The EnvironMonitor board connected to the real and electrically simulated environmental inputs. Labels identify the water-level, anemometer, rain-gauge and thermohygrometer connections used in the two validation cases.

Figure 18 shows the EnvironMonitor board with the simulated and real sensor connections. Two test cases were considered. In the first case, the rain simulation frequency was set to 0.01 Hz, corresponding to one tipping event every 100 s and therefore to 7.2 mm/h. The water-level current was set to 12 mA, corresponding to 15 m. The wind-speed rotor was not set in motion; the wind direction was approximately aligned with the mast direction, and the thermohygrometer measured room conditions. In the second case, the rain simulation frequency was set to 1 Hz, corresponding to 720 mm/h, and the water-level current was set to 20 mA, corresponding to 30 m. The wind direction was adjusted to a near-opposite direction, and the local humidity was intentionally increased by breathing on the thermohygrometer. In both cases, the printed values were consistent with the imposed or expected conditions, as quantified below.

Table 3 summarises the corresponding numerical outputs. In the first test, EnvironMonitor returned a wind speed of 0.00 m/s, a wind direction of 15°, a rainfall rate of 7.74 mm/h, an air temperature of 26.75 °C, a relative humidity of 61.81% and a water level of 14.99 m. In the second test, the measured outputs were 0.00 m/s, 180°, 713.58 mm/h, 26.84 °C, 82.58% and 29.89 m, respectively.

The water-level conversion showed deviations of less than 0.4% in both tests; the zero wind-speed condition was also correctly detected, and the measured wind directions were consistent with the manually imposed vane orientations. The deviation in the measured wind direction recorded during the first test corresponds to an absolute difference of approximately 5–7°, remaining within the range typically considered acceptable for monitoring ordinary bridges. The increase in relative humidity was also captured with acceptable precision. Regarding rainfall-rate deviations, these reached +7.50% and −0.89% for the low- and high-rate simulations, respectively. At the low simulated rainfall rate, the +7.50% relative difference corresponds to an absolute difference of 0.54 mm/h; because the rain channel derives the rate from discrete tipping events and elapsed time, finite timing/pulse quantisation has a proportionally larger influence at low rates.

Overall, these tests verified the input conditioning, conversion, acquisition and communication functions of EnvironMonitor for its intended uses. It should be noted, however, that the electrical simulation does not reproduce field effects acting on the physical radar and rain-gauge measurements, such as water-surface turbulence, splashing, floating debris, condensation or icing, changes in reflection conditions and mounting geometry. Accordingly, the present tests are intended to validate the electronic acquisition and conversion chain under controlled inputs; the complete sensor-to-environment performance will be assessed during the forthcoming in situ tests on full-scale bridge case studies under real environmental conditions. Similarly, only two operating points were tested; full-range calibration and repeatability will be included in subsequent field validation.

## 7. Power-Consumption Estimates, Scalability and Cost Analysis

### 7.1. Power-Consumption Estimates

The power demand of the proposed system was estimated using the worst-case current consumption values reported in the component datasheets and the nominal efficiencies of the DC–DC converters. The components of each BridgeWatch node draw an estimated total current of 32.32 mA from the 3.3 V supply. At 80% efficiency for the 12 V-to-3.3 V converter, this corresponds to about 11.11 mA drawn from the 12 V battery, equivalent to 0.133 W per node. The low-voltage electronics of EnvironMonitor draw approximately 37.65 mA at 3.3 V, corresponding to 12.94 mA from the 12 V supply.

The Raspberry Pi represents the main contribution to the power budget. Assuming, conservatively, a current demand of 2 A at 5 V and an efficiency of 88% for the 12 V-to-5 V converter, its equivalent current demand from the battery is approximately 0.947 A. Including the maximum 20 mA loop current of the water-level sensor, the complete EnvironMonitor, Raspberry Pi and water-level-sensor branch requires approximately 0.980 A at 12 V. Consequently, a representative configuration comprising ten BridgeWatch nodes and one fully equipped EnvironMonitor unit has an estimated total current demand of approximately 1.09 A at 12 V, corresponding to 13.1 W or approximately 314 Wh per day. The selected 12 V, 80 Ah battery has a nominal stored energy of 960 Wh. At an estimated continuous current demand of 1.09 A, this corresponds to a theoretical autonomy of approximately 73 h in the absence of photovoltaic recharge. This value assumes that the entire nominal battery capacity is available. If only 50% of the nominal capacity is conservatively considered usable, the corresponding autonomy decreases to approximately 37 h.

In this context, the photovoltaic panel is intended to replenish the daily energy demand and support long-term autonomous operation, subject to site-specific solar availability. A preliminary photovoltaic-panel rating can be estimated from the daily energy demand as $P_{PV}=E_{day}/(H_{PSH}\eta_{system})$, where $H_{PSH}$ is the number of equivalent peak-sun hours per day and $\eta_{system}$ accounts for charging, conversion, orientation and temperature-related losses. Assuming (again, conservatively) four equivalent peak-sun hours per day and an overall efficiency of 75%, the minimum calculated photovoltaic power is approximately 105 W. A nominal panel rating of approximately 150 W would therefore provide a reasonable preliminary design margin under these assumptions. For less favourable winter conditions, such as two equivalent peak-sun hours per day, a panel in the approximate range of 250–300 W would be more appropriate.

Note that these power figures are design estimates derived from datasheets and converter efficiencies. In addition, the Raspberry Pi-based configuration cannot be directly characterised as an ultra-low-power system. In the representative ten-node budget, the Raspberry Pi branch accounts for approximately 87% of the estimated 13.1 W total, and the battery/PV sizing is a theoretical engineering estimate; actual long-term endurance will be validated via future field tests.

### 7.2. Practical Network Scalability, Data Volume and Maintenance

The experimentally validated configuration comprised four BridgeWatch nodes, whereas the power and cost analysis used a ten-node arrangement. Both represent smaller or larger design scenarios for different typical short- or long-span bridges rather than an experimentally determined maximum node count. At the software level, the Raspberry Pi manages BridgeWatch nodes through individual identifiers, while each board samples locally and temporarily stores the measurements before responding to the coordinator. Adding nodes, therefore, does not directly modify the local sampling interval, although it increases communication and data management demands on the shared RS485 bus.

At the physical layer, the selected THVD1400 RS485 transceiver is a 1/8-unit-load device, corresponding to a theoretical electrical limit of up to 256 transceiver nodes and a maximum specified data rate of 500 kbps. These component-level ratings should not be interpreted as the demonstrated maximum size of the complete BridgeWatch network: The practical application-level limit will be lower and will depend on bus length and topology, UART baud rate, protocol overhead, polling frequency, local buffer capacity, Raspberry Pi processing time and the one-second transfer/re-synchronisation interval. For practical implementation, monitoring schemes commonly rely on 9–10 channels per span [43]. Configurations with approximately 40 channels are also typically used for continuous multi-span bridges or highly instrumented single spans (see, e.g., [44,45]).

Accordingly, the present study demonstrates four-node operation and evaluates a ten-node deployment scenario. Determining the maximum practical network size will require dedicated scalability testing, but no theoretical constraint prevents upscaling from 4 or 10 nodes to quantities of the same order of magnitude as in the provided examples and similar applications.

Raw-data volume also becomes relevant as the network grows. At 256 Hz and 59 s of effective acquisition per minute, each BridgeWatch node produces 15,104 frames per acquisition cycle. Considering the eight 32-bit quantities stored for each structural frame, this corresponds to approximately 0.483 MB/min per node in binary-equivalent form, or approximately 0.696 GB/day per node for continuous operation. A ten-node configuration would therefore generate approximately 6.96 GB/day before accounting for text-format overhead. Long-term operation consequently favours binary or compressed storage, file rotation, scheduled acquisition, and in particular, an envisaged Raspberry Pi edge-processing strategy, whereby reduced modal and diagnostic features are transmitted routinely, while complete time histories are retained locally to be deleted or transferred selectively at a later stage.

From a maintenance perspective, the principal field-service items are expected to include inspection of the battery and photovoltaic charging system, cable and connector integrity, cable glands and enclosure sealing, local-storage health, remote communication status and periodic checks for sensor drift or calibration changes. This aligns with current best practices in SHM systems upkeep. In particular, the proposed wired topology avoids individual batteries at each structural node but places greater importance on cable integrity and the central power system. Remote diagnostics of node availability, battery voltage, file generation and communication errors should therefore form part of the future operational deployment software, which is under construction and not directly assessed in this work.

### 7.3. Cost Analysis

Cost was a primary design constraint in this research work. As is widely known, a key limitation to the widespread use of SHM is the economic incompatibility of expensive setups with the large number of road and rail assets deemed at risk. From this perspective, the BridgeWatch node has an estimated cost of approximately €178, including the PCB, electronic components, PT100 sensor and protective box. The EnvironMonitor board has an estimated cost of approximately €310 without the rain gauge and water-level sensor, and approximately €1500 with all environmental sensors included. The additional system components, including the enclosure for EnvironMonitor/Raspberry/battery, cable, Raspberry Pi, battery and solar panel, were purchased at approximately €785.

The laboratory prototype considered here includes four BridgeWatch nodes and one EnvironMonitor, excluding the rain gauge and water-level sensor, for an estimated cost of €1800. A complete monitoring configuration suitable for a typical Italian two- or three-span bridge, including ten BridgeWatch nodes, one fully equipped EnvironMonitor unit and the auxiliary components, is estimated to cost approximately €4100. These values are updated to March 2024 and exclude taxes, some custom mechanical supports, the bridge pole for environmental sensors and additional installation costs. Nevertheless, the result supports the premise that a dedicated, low-cost hardware platform can reduce the barrier to continuous bridge monitoring, particularly for local infrastructure (e.g., municipality- or province-level bridges) where budgets are limited and full commercial SHM systems are economically difficult to justify.

In conclusion to this cost analysis, note that all the monetary values reported here are hardware procurement/Bill-of-Materials estimates, not total lifecycle costs. They exclude manual assembly, calibration services or commercial margins, as well as operating costs, such as recurring communications/server costs and long-term maintenance. The “low-cost” label therefore refers to the custom sensing/acquisition hardware and to the order-of-magnitude comparison in Table 4, not to a complete installed turnkey SHM service.

## 8. Mechanical Integration for Future Outdoor Deployment

**Figure 19:**
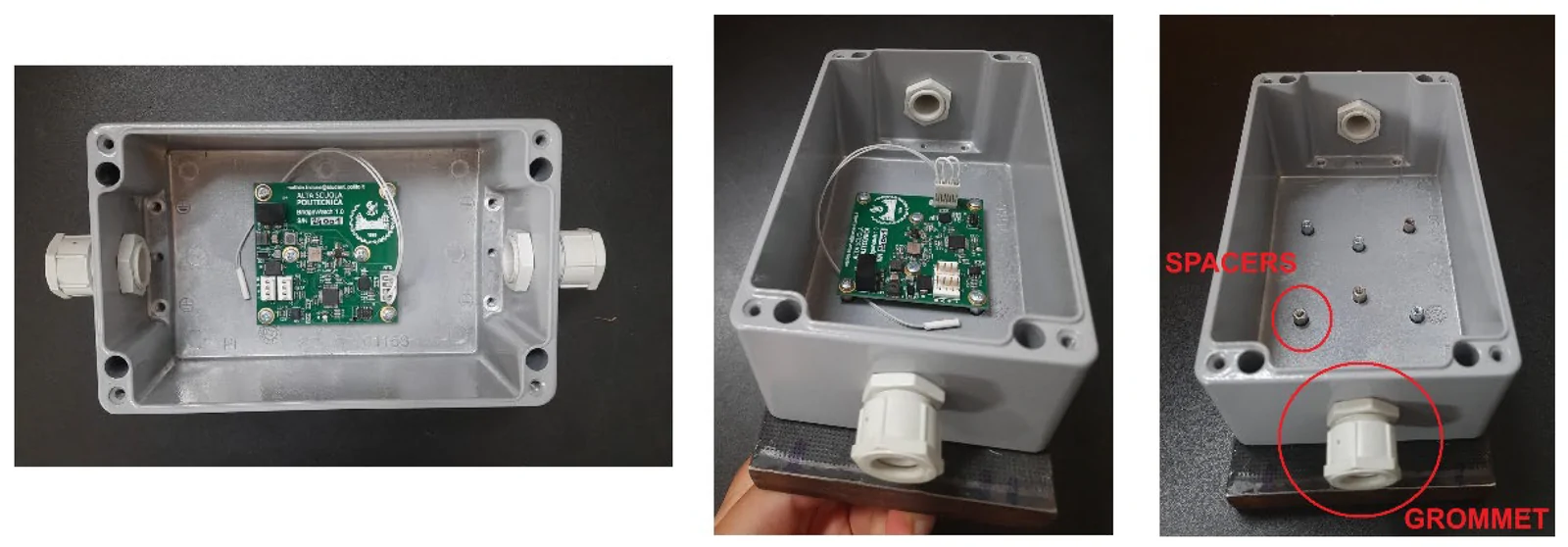
View of the open protective enclosure of the prototype BridgeWatch, showing the internal mounting plate, spacers, grommet and installed board.

To avoid any disturbance from case−sensor interactions, all laboratory tests described in this article were performed directly using the BridgeWatch and EnvironMonitor boards. Nevertheless, outdoor bridge monitoring requires mechanical and hydraulic protection, cable management and a minimally intrusive mounting strategy. For these reasons, both BridgeWatch and EnvironMonitor were designed to be attached to a metal plate mounted inside a commercial IP66 protective enclosure. The metal plate can be glued to the bridge surface, limiting the need for invasive work. The enclosure uses cable glands and internal spacers to protect the PCB while allowing the communication and power cable to enter the box. Figure 19 shows the open enclosure with the board mounted inside it.

However, at its current stage, the prototype requires additional work before outdoor deployment. The complete field system is intended to use a shielded outdoor cable, a battery and a photovoltaic panel. The protective boxes and cable glands are central to this transition from laboratory prototype to field-ready monitoring node. Accordingly, “field deployable” and enclosure-related statements throughout this paper refer to design provisions, not to experimentally demonstrated long-term outdoor performance.

As mentioned before, the present work is specifically intended to validate the sensing, acquisition, synchronisation and environmental-interface functions under controlled laboratory conditions; it does not constitute a long-term field validation of the complete system. Consequently, communication availability over extended cable runs, long-term enclosure and connector performance, electromagnetic compatibility under field conditions, seasonal photovoltaic yield, battery ageing, sensor drift and timing stability will be assessed under operational bridge conditions. In situ tests on full-scale bridge case studies are planned as the next validation stage and will be conducted shortly.

## 9. Discussion

The results demonstrate experimentally that the proposed sensing network can coherently acquire structural and environmental data across several laboratory scenarios. The preliminary test verified communication, synchronism and acquisition continuity across four BridgeWatch boards. In the shaker tests, the BridgeWatch board correctly identified the imposed sinusoidal excitation frequencies; tests at different driving frequencies confirmed that the accelerometer and acquisition chain can recover imposed dynamic inputs with negligible frequency error over the tested range. The boards were subsequently tested on a scaled building model, which is more representative of SHM applications because it was used to identify natural frequencies from ambient vibrations and free-decay responses rather than simply recover a known input. In these tests, the first six vibration modes were consistently identified. The estimated natural frequencies also showed good agreement with the reference values reported in previous studies. Finally, a direct comparison with synchronous reference accelerometers also showed that BridgeWatch captured the relevant spectral peaks with comparable SNRs.

The environmental tests confirmed that EnvironMonitor can handle both real sensor inputs and simulated sensor outputs with different electrical characteristics: pulse signals for rainfall, 4–20 mA current for water level, potentiometric voltage for wind direction, pulse counting for wind speed and I2C digital data for air temperature and humidity. The EnvironMonitor tests produced environmental readings aligned with the imposed rainfall-rate and water-level scenarios. This multi-sensor integration is important for bridge monitoring because environmental and hydrological conditions can mask or mimic structural changes. Recording these quantities, together with the structural response, is indeed essential for damage assessment based on a correct interpretation of scour-related risk scenarios.

A practical use of the multi-domain measurements can be formulated through environmental normalisation. Let $y_{i}(t)$ denote a monitored structural feature, such as a natural frequency or quasi-static inclination, and let e(t) contain the simultaneously measured temperature, humidity, wind, rainfall and water-level variables. During an initial reference period in which the structure is assumed to be healthy, a statistical or regression baseline ${\hat{y}}_{i}(t)=g_{i}[e(t)]$ can be identified to represent the expected environmental dependence of the structural feature. Subsequent monitoring can then be based on the residual $r_{i}(t)=y_{i}(t)-{\hat{y}}_{i}(t),$ rather than directly on the raw feature, reducing false indications associated with normal environmental variability. Similar approaches have been shown to be viable in the literature; see, e.g., [46].

The climatic-cell experiment already demonstrates the physical motivation for such compensation, because the accelerometer output showed a measurable temperature dependence. Rainfall and water level play complementary roles: rather than being merely nuisance variables to remove, they can serve as event/context variables that increase monitoring priority during high-water conditions and aid interpretation of concurrent changes in pier rotation or modal properties potentially linked to foundation scour. Note that the present apparatus does not directly measure scour depth, and no environmental-normalisation or scour-detection algorithm is claimed as experimentally validated here. The forthcoming full-scale bridge campaigns will provide the coupled structural/environmental datasets required to implement and validate these fusion strategies.

Summarising all these experimental results, the focus was on a new hardware design of a multi-domain sensor network that can provide reliable structural and environmental data for subsequent model-based or data-driven processing, with a coordinated combination of synchronised dynamic and quasi-static structural measurements alongside environmental and hydrological sensing and field-oriented power and communication provisions. The validation should therefore be interpreted at distinct evidence levels. The impulse and shaker tests verify acquisition, timing alignment at integer-sample resolution and frequency recovery; the scaled benchmark verifies extraction of modal features and consistency with an independent acquisition chain (but not absolute sensor calibration); the climatic-cell test quantifies temperature-dependent acceleration offset; and the EnvironMonitor tests verify conditioning/conversion at selected input points.

### Quantitative Comparison with Representative SHM Systems

To position the proposed platform quantitatively, Table 4 compares BridgeWatch with representative low-cost research systems and representative commercial/reference configurations. Values are taken from the cited publications or from hardware specifications used in the present design; therefore, this table is a specification- and system-level comparison, not a claim that all systems were tested side by side.

The values in Table 4 should be interpreted according to their respective system boundaries. The approximately €4100 estimate for the present system includes ten structural nodes, a fully equipped environmental node, a Raspberry Pi, cabling, a battery, a photovoltaic supply and auxiliary hardware, whereas several published costs refer only to structural sensing and acquisition. The comparison therefore illustrates orders of magnitude and performance–cost trade-offs rather than a strict procurement comparison. Synchronisation is a system-level characteristic rather than an intrinsic accelerometer property, and commercial specification data are not presented as identical-condition experimental results. The only same-structure comparison carried out in this study is the model-building test against the Moni2BSafe reference acquisition chain, discussed previously in more detail.

However, compared with existing commercial SHM solutions, the proposed system offers a more flexible and cost-effective alternative for bridges where dense instrumentation is desirable, but budget constraints limit adoption of proprietary monitoring platforms.

On the one hand, commercial systems typically provide mature hardware and integrated software environments, which are often expensive, closed (thus, difficult to integrate), and less adaptable to project-specific sensor layouts or research-oriented data processing. In contrast, the proposed architecture is based on custom, low-cost PCBs, standard communication protocols and open acquisition logic that can be modified, extended and replicated to suit the bridge geometry and monitoring objectives. Combined with the relatively low cost, this makes the proposed system suitable for distributed deployment with multiple structural nodes.

Moreover, the system not only acquires vibration data, as many other research prototypes reported in the current scientific literature do. The proposed apparatus also integrates measurements of quasi-static sensing capability, surface temperature, wind, rainfall, air temperature, humidity and water level into a single, synchronised framework. This multi-sensor capability provides a practical advantage for bridge SHM and scour-related monitoring, since dynamic and static features can be analysed together, rather than separately, and interpreted alongside environmental and hydraulic boundary conditions. This choice incorporates engineering knowledge and domain expertise into the SHM system deployment, enabling more cost-efficient monitoring by exploiting the features expected to be most damage-sensitive at different locations (e.g., rotations at piers and vibrations along the span).

## 10. Conclusions and Future Work

In this work, a low-cost multi-sensor acquisition platform for Structural Health Monitoring and scour-related environmental monitoring was designed, implemented and functionally validated under controlled laboratory conditions. The hardware comprises BridgeWatch structural nodes and an EnvironMonitor environmental/hydrological interface, coordinated through a Raspberry Pi and connected via RS485/RS232 links.

The central contribution is the integration of structural, temperature, meteorological and hydrological acquisition in one configurable architecture that can supply later SHM, environmental-normalisation and scour-risk interpretation. The specific key outcomes are as follows:

- The laboratory campaign confirmed the main acquisition functions. BridgeWatch detected common impact events across four boards and recovered the commanded shaker frequencies with small FFT-bin differences from the nominal setpoints. On the scaled benchmark, the first six modal frequencies were identified, and the spectra were consistent with the separate Moni2BSafe acquisition chain. These comparisons demonstrate functional modal data acquisition.
- The climatic-cell test quantified a modest temperature dependence of the *z*-axis acceleration output. Furthermore, EnvironMonitor correctly processed selected real and electrically simulated input conditions; these tests validate signal conditioning and conversion at the tested points.
- The resulting platform is modular and uses comparatively low-cost custom structural nodes.

Future development revolves around in situ validation, with field tests for long-term bridge monitoring. This will possibly be organised in three targeted phases. Phase 1 will consist of the in situ deployment on full-scale operational bridges, including quantitative checks of long-cable RS485 reliability, enclosure performance, assembled-node noise, pre-resynchronisation timing drift, real power consumption, battery/photovoltaic endurance and full sensor-chain environmental behaviour. Phase 2 will address multi-sensor fusion and interpretation, including controlled inclination calibration, accelerometer–gyroscope fusion and environmental normalisation of structural features. Phase 3 will address embedded data reduction: lightweight FFT/feature extraction will be ported and benchmarked on the Raspberry Pi, while computationally heavier modal-identification routines can remain server-side initially; the feasibility of a lower-power coordinator can also be assessed. These full-scale in situ tests are planned to follow shortly, and their results will be released following the present laboratory-focused paper.

## Figures and Tables

**Table 1:** Summary of shaker-test frequency- and amplitude-identification results. Nominal input values were defined according to the shaker setpoints.

Table	Nominal Input Amplitude [g]	BridgeWatch Identified Fundamental Amplitude [g]	Signed Amplitude Difference from Nominal Setpoint [%]	Nominal Input Frequency [Hz]	BridgeWatch Identified Frequency Peak [Hz]	Signed Frequency Difference from Nominal Setpoint [%]	Notes
(i)	0.500	0.503	+0.60	5.00	5.00	0.00	Frequency peak recovered; low-frequency shaker waveform contains visible distortion/harmonics.
(ii)	1.000	0.991	−0.90	10.00	10.02	+0.17	Clear dominant spectral peak; amplitude value is relative to the nominal command setpoint.
(iii)	1.000	0.951	−4.90	20.00	20.02	+0.08	Clear dominant spectral peak; amplitude value is relative to the nominal command setpoint.
(iv)	1.000	0.918	−8.20	50.00	50.07	+0.14	Frequency recovered; frequency-dependent amplitude attenuation is not used as an accuracy calibration.
(v)	1.000	0.854	−14.60	100.00	100.12	+0.12	Frequency recovered despite sparse samples per cycle.

**Table 2:** Natural frequencies identified on the scaled building model, compared with earlier benchmark values and the separate Moni2BSafe acquisition chain.

Identified Mode Number	Previous Tests (Dragonetti et al. [41], Table 9) [Hz]	Reference (Moni2BSafe) [Hz]	This Study’s (BridgeWatch) [Hz]	Mode Interpretation
1	2.94	2.58	2.60	First flexural mode along Y
2	5.96	5.23	5.24	First flexural mode along X
3	7.70	7.28	7.30	First torsional mode
4	8.89	8.01	8.30	Second flexural mode along Y
5	13.65	13.05	13.06	Third flexural mode along Y
6	26.43	25.44	25.50	Second flexural mode along X

**Table 3:** Comparison between the imposed or expected environmental conditions and the outputs returned by EnvironMonitor.

Test Case	Quantity Measured	Imposed or Expected Value	EnvironMonitor Output	Signed Relative Difference [%]
1	Wind speed [m/s]	0.00	0.00	0.00
1	Wind direction [°]	~0–10	15	~0.00
1	Rainfall rate [mm/h]	7.20	7.74	+7.50
1	Air temperature [°C]	27.00	26.75	−0.93
1	Relative humidity [%]	60.00	61.81	+3.02
1	Water level [m]	15.00	14.99	−0.07
2	Wind speed [m/s]	0.00	0.00	0.00
2	Wind direction [°]	~170–180	180	~0.00
2	Rainfall rate [mm/h]	720.00	713.58	−0.89
2	Air temperature [°C]	27.00	26.84	−0.59
2	Relative humidity [%]	80.00	82.58	+3.23
2	Water level [m]	30.00	29.89	−0.37

**Table 4:** Representative performance–cost comparison of the proposed platform with low-cost research systems and commercial/reference instrumentation. The comparison includes the low-cost research systems discussed in Refs. [18,19,20] and the recent offline synchronised DAQ architecture of Alandí et al. [39].

Scheme	Structural Sensor Performance	Sampling/ Synchronisation	Communication/ Power	Indicative Cost/ Validation
BridgeWatch (this work)	ADXL355, ±2 g; 3.9 µg/LSB; 22.5 µg/√Hz; zero-g offset temperature coefficient ≤ 0.15 mg/°C.	256 Hz in this study (ADXL355 ODR selectable up to 4 kHz); periodic 60 s re-alignment; zero integer-sample lag in the impulse test at 3.906 ms resolution.	Wired RS485 structural bus; ≈0.133 W equivalent per BridgeWatch node; central 12 V battery with photovoltaic recharge.	≈€178 per BridgeWatch node; ≈€4100 representative ten-node multi-domain installation. Laboratory validation plus same-structure modal comparison.
Muttillo et al. [18]	Custom triaxial accelerometer nodes; structural-vibration focused.	Datalogger-level acquisition synchronisation; local node memory.	Wired RS485 to customised datalogger; power autonomy not the principal validated contribution.	Cost not reported on a directly comparable basis; laboratory structural-damage-indicator validation.
WindNode, Zanelli et al. [19]	MEMS accelerometric node for low-frequency structural vibration.	On-board FFT and configurable acquisition; wireless-node timing strategy.	BLE communication; low-power duty cycling; Li-Po battery with small photovoltaic energy harvesting.	Cost not reported on the same system boundary; laboratory calibration plus field validation under harsh conditions.
Villacorta et al. [20]	ADXL355, ±2 g; 3.9 µg/LSB; ≈25 µg/√Hz.	Up to 4 kHz; myRIO acquisition; dedicated synchronisation line.	Wired acquisition; power reported for acquisition hardware rather than autonomous field operation.	≈€928 reported for six low-cost accelerometers, DAQ and cabling; laboratory validation against commercial reference instrumentation.
LARA, Komarizadehasl et al. [21,22]	Low-cost multi-MEMS acceleration node; reported noise density ≈ 51 µg/√Hz.	333 Hz; timestamp/post-synchronisation strategy.	Arduino/Raspberry Pi architecture with wireless control.	Low-cost research prototype; validated against a commercial high-precision sensor on a footbridge.
Hasani et al. [23]	ADXL355-based wireless MEMS node; 20-bit sensor output.	Selectable 62.5–500 Hz; wireless synchronisation and preprocessing.	Wi-Fi with 4G backhaul; battery-powered long-term architecture.	Cost not reported in a directly comparable system boundary; field-tested using 30 sensors on a concrete arch bridge.
Hidalgo-Fort et al. [24]	ADXL355-based modular IoT sensor node; 20-bit/≈3.8 µg resolution.	31.25 Hz used for bridge modal monitoring; GPS-assisted node synchronisation.	NB-IoT; FreeRTOS/edge Random Decrement processing; measured power characterisation and long-autonomy duty cycle.	≈€175 per node; pilot-structure and Eduardo Torroja bridge field validation against commercial systems.
Di Nuzzo et al. [25]	MEMS-based long-term modal-monitoring node.	Sampling/synchronisation tailored to long-term modal monitoring.	NB-IoT communication; low-power/energy-neutral concept with photovoltaic supply.	Cost not reported on a directly comparable basis; long-term monitoring architecture.
Alandí et al. [39]	ADXL355 inertial node plus separate 24-bit ADS1220 displacement node.	1000 Hz inertial sampling; DS3231MZ TCXO and 1 Hz hardware PPS for offline synchronisation.	Autonomous local PSRAM + microSD storage; no continuous acquisition network; per-node battery power.	Cost not reported on the same boundary; full-scale field campaign with 53 physical DAQ nodes covering 118 measurement points.
PCB Piezotronics 393B12 [47]	High-sensitivity ICP accelerometer; 10 V/g; 0.5 g pk range; 8 µg rms broadband resolution; spectral noise 1.30 µg/√Hz at 1 Hz and 0.32 µg/√Hz at 10 Hz; specified phase response ±5° from 1 to 1000 Hz.	Analog sensor; acquisition sampling and inter-channel synchronisation are determined by the external DAQ. Sensor phase response specified to ±5° over 1–1000 Hz.	Wired ICP/IEPE interface; 18–30 VDC excitation with a 2–20 mA constant current supply; no autonomous node-level power system.	≈US$908 per sensor in the manufacturer’s listing at the access date; external DAQ and cabling not included.
Commercial reference reported by Villacorta et al. [20]	KS76C.100 IEPE accelerometer: ±60 g; 100 ± 5 mV/g; ≈3 µg/√Hz.	DS-SIRIUS 24-bit synchronous DAQ; up to 200 kS/s/channel; reported DAQ dynamic range up to 160 dB.	Wired synchronous commercial DAQ; ≈1 W/channel reported for DAQ input modules.	≈€9050 reported for six sensors, DAQ and cabling; research-grade commercial reference.

## Data Availability

The PCB schematics and MATLAB routines used for the reported analyses are provided in Supplementary Materials. Gerber files, firmware source and representative sample records are available from the corresponding author upon reasonable request.

## Supplementary Materials

The following supporting information can be downloaded at: https://www.mdpi.com/article/10.3390/s26185957/s1, Figure S1: BridgeWatch first schematic section, including the STM32 microcontroller, ADXL355 accelerometer, I3G4250D gyroscope, and related digital interfaces; Figure S2: BridgeWatch second schematic section, including the power supply, RS485 transceiver, RTD-to-digital conversion chain, LED, and auxiliary connectors; Figure S3: EnvironMonitor first schematic section, including the microcontroller, RS232 interface, power supply, and battery-charge monitoring circuitry; Figure S4: EnvironMonitor second schematic section, intended for the conditioning and protection of environmental sensor inputs; Figure S5: EnvironMonitor third schematic section, intended for the 12 V-to-5 V conversion and the USB-A output used to supply the Raspberry Pi; Figure S6: Flowchart of the Raspberry Pi acquisition routine and menu-driven control logic.
