# Supplementary material for: Design, Development, and Laboratory Validation of a Low-Cost Multi-Sensor System for Bridge Structural Health Monitoring with Scour-Related Environmental Sensing
Source: Sensors (Basel). 2026 Sep 20;26(18):5957. doi: 10.3390/s26185957 (PMC13611220; doi:10.3390/s26185957)
Supplement: Supplementary file 1 [file sensors-26-05957-s001.zip › sensors-4504628-supplementary.pdf]

Supplementary Materials

Section A

BridgeWatch and EnvironMonitor Schematics and Main Circuit Blocks

The BridgeWatch schematics are shown in Figures S1 and S2. Figure S1 shows the microcontroller and digital sensor section, with the accelerometer and gyroscope connected by SPI. This choice provides short-range, high-speed, low-power board-level communication and minimises complexity for synchronous acquisition. Interrupt and data-ready lines are routed to the microcontroller to manage sampling and sensor status.

Figure S2 includes the 12 V input, DC–DC converter, RS485 transceiver, RTD-to-digital converter, programming/debug connector and auxiliary circuitry. The RS485 interface was adopted because it supports differential signalling, improved noise immunity and multi-drop bus operation over longer cables. This is essential for the intended bridge installation, in which several structural nodes should be distributed along the deck span and near the supports.

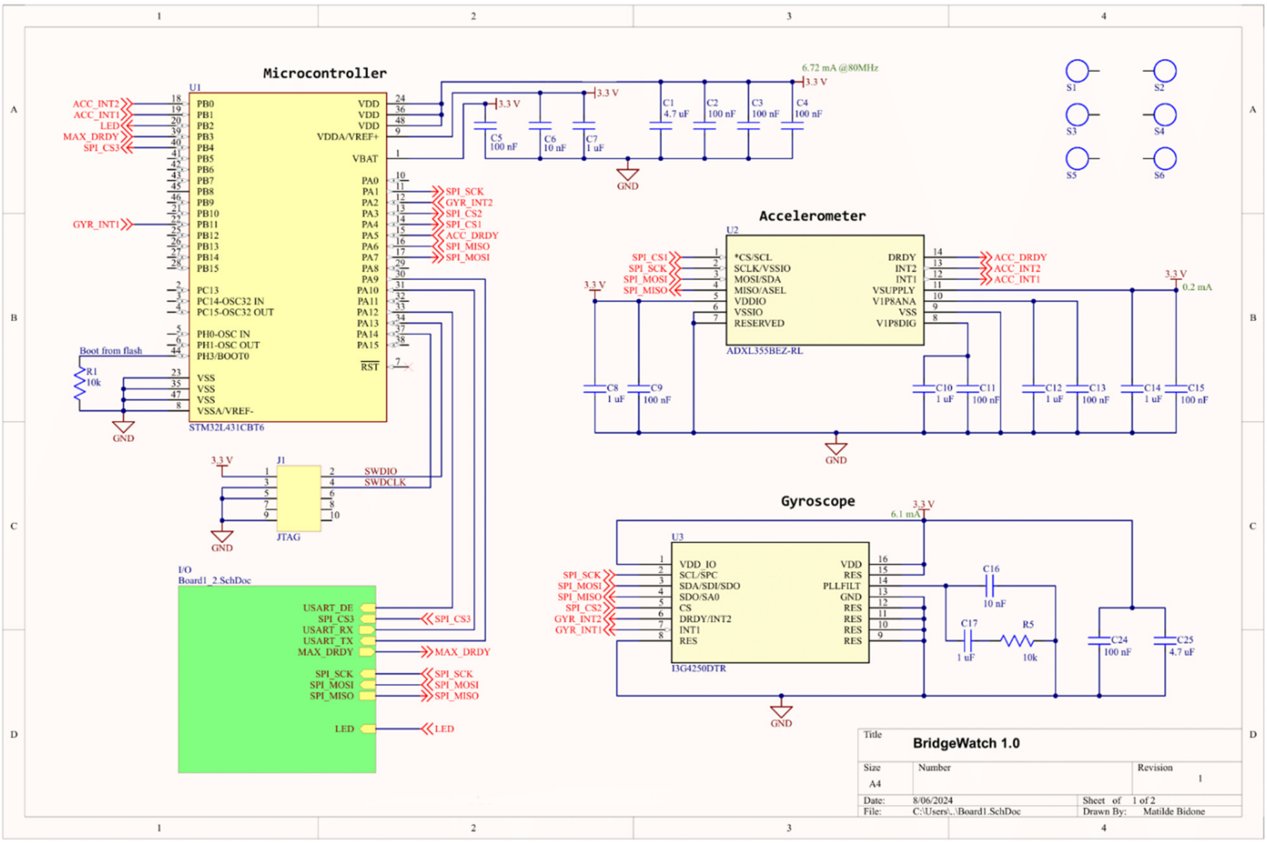

Figure S1. BridgeWatch first schematic section, including the STM32 microcontroller, ADXL355 accelerometer, I3G4250D gyroscope and related digital interfaces.

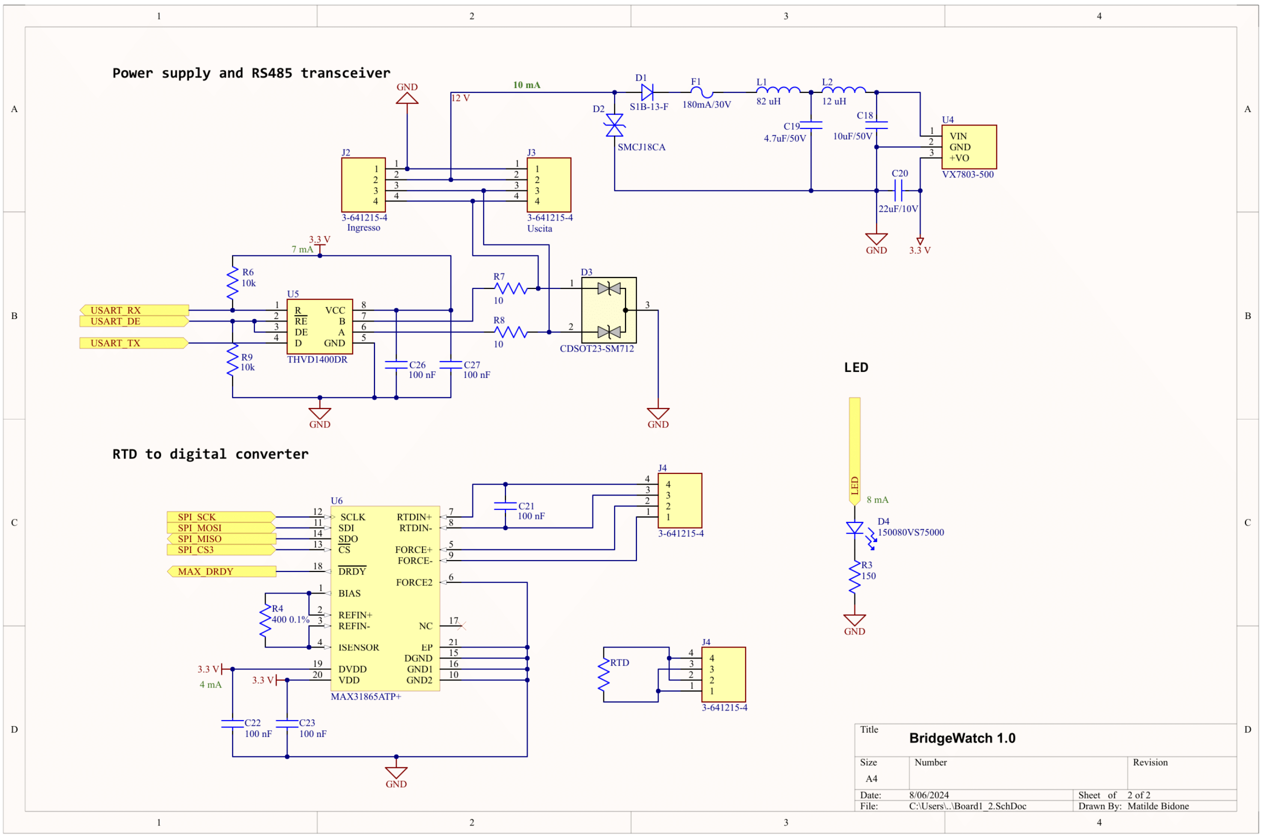

Figure S2. BridgeWatch second schematic section, including the power supply, RS485 transceiver, RTD-to-digital conversion chain, LED and auxiliary connectors.

The EnvironMonitor schematics are shown in Figures S3–S5.

The first section (Figure S3) includes the microcontroller, the 12 V-to-3.3 V conversion, the 12 V-to-5 V conversion for powering the Raspberry Pi, RS232 communication and battery charge monitoring. Unlike the structural nodes, EnvironMonitor uses a one-to-one RS232 link to the Raspberry Pi because only one environmental board is required in the reference configuration.

Figure S4 shows the signal-conditioning circuitry for the external environmental sensors. Each external input includes protection and conditioning elements to limit the effects of electromagnetic interference, voltage spikes and electrostatic discharge. The water-level sensor current is converted and buffered before ADC conversion, the wind-direction potentiometric output is conditioned before entering the ADC, and digital/pulse inputs are protected and debounced where required.

Finally, Figure S5 focuses on the converter and USB-A output used to supply the Raspberry Pi from the same 12 V power system. Integrating this supply into EnvironMonitor reduces the amount of auxiliary hardware required in the field enclosure and keeps the system architecture compact.

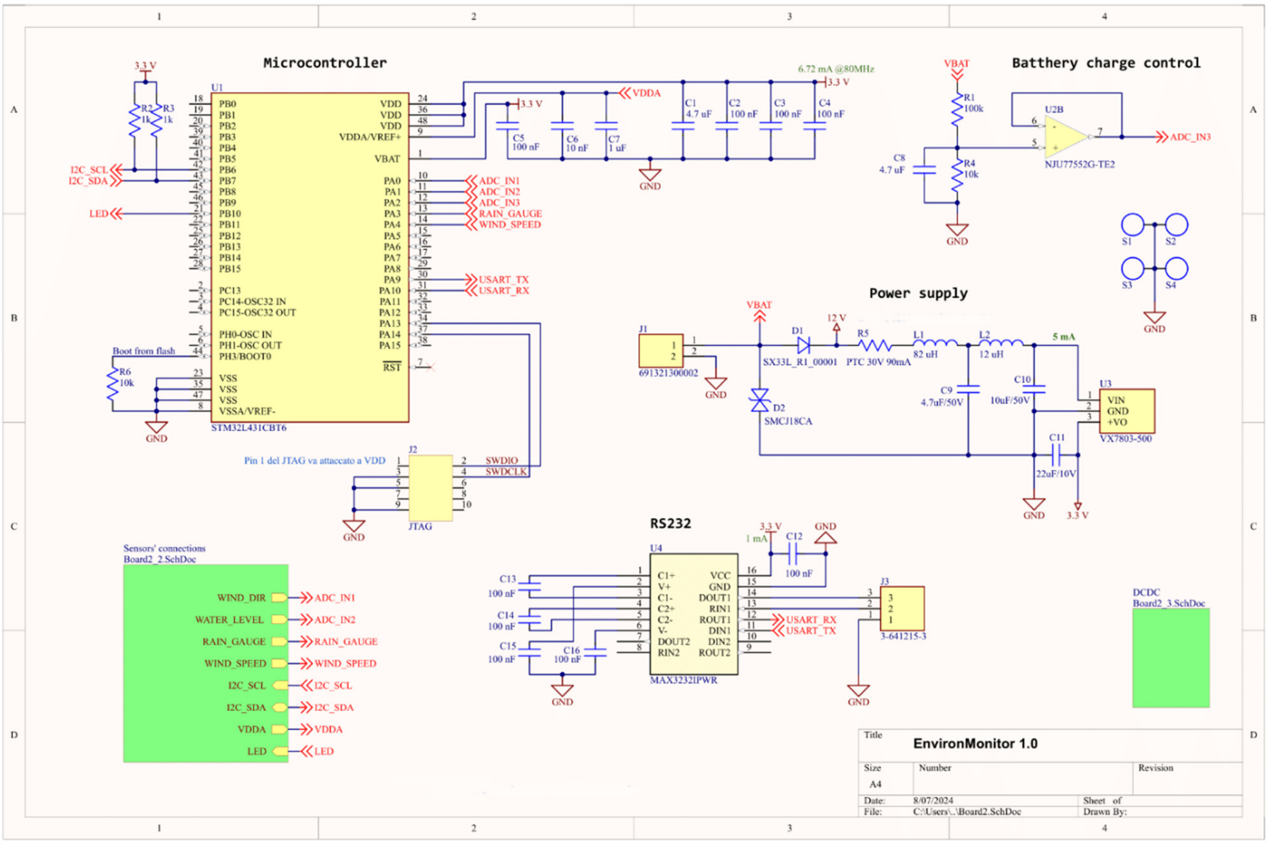

Figure S3. EnvironMonitor first schematic section, including the microcontroller, RS232 interface, power supply and battery-charge monitoring circuitry.

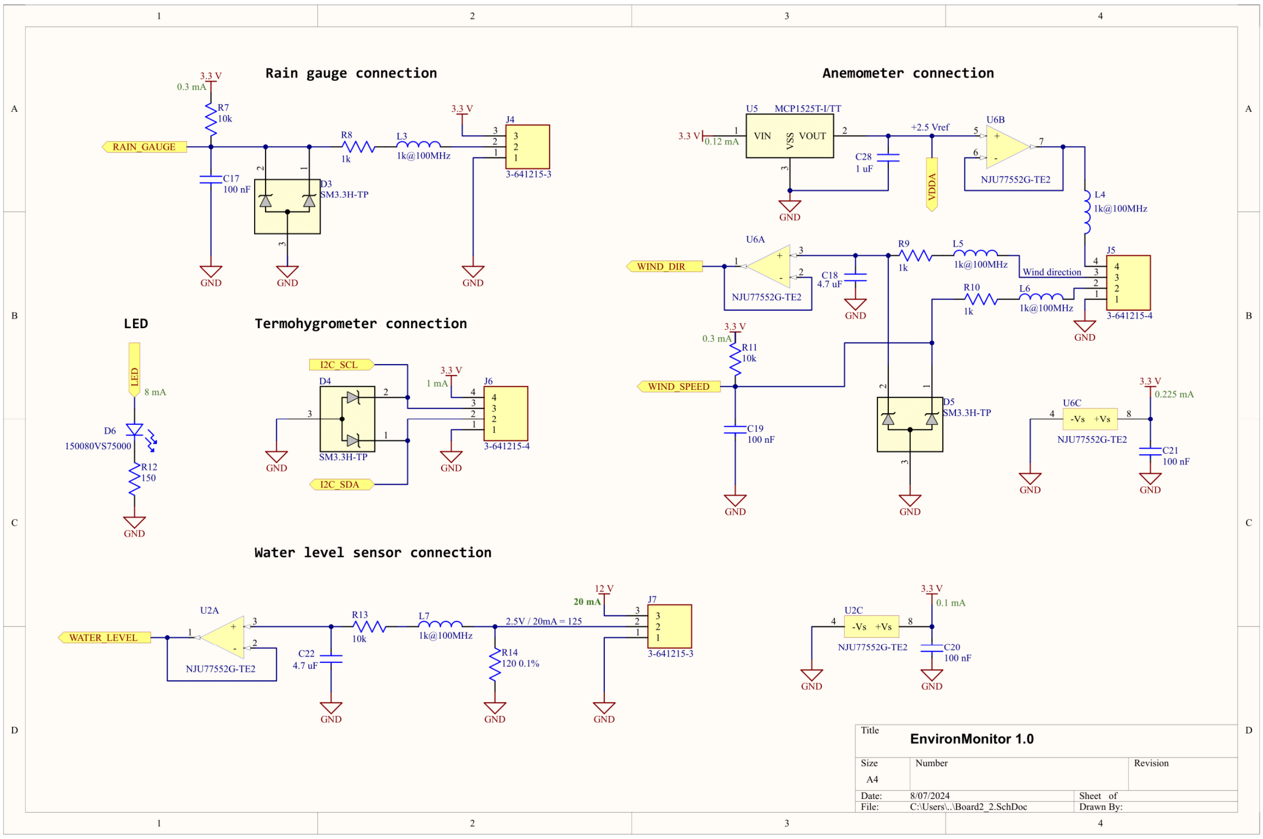

Figure S4. EnvironMonitor second schematic section, intended for the conditioning and protection of environmental sensor inputs.

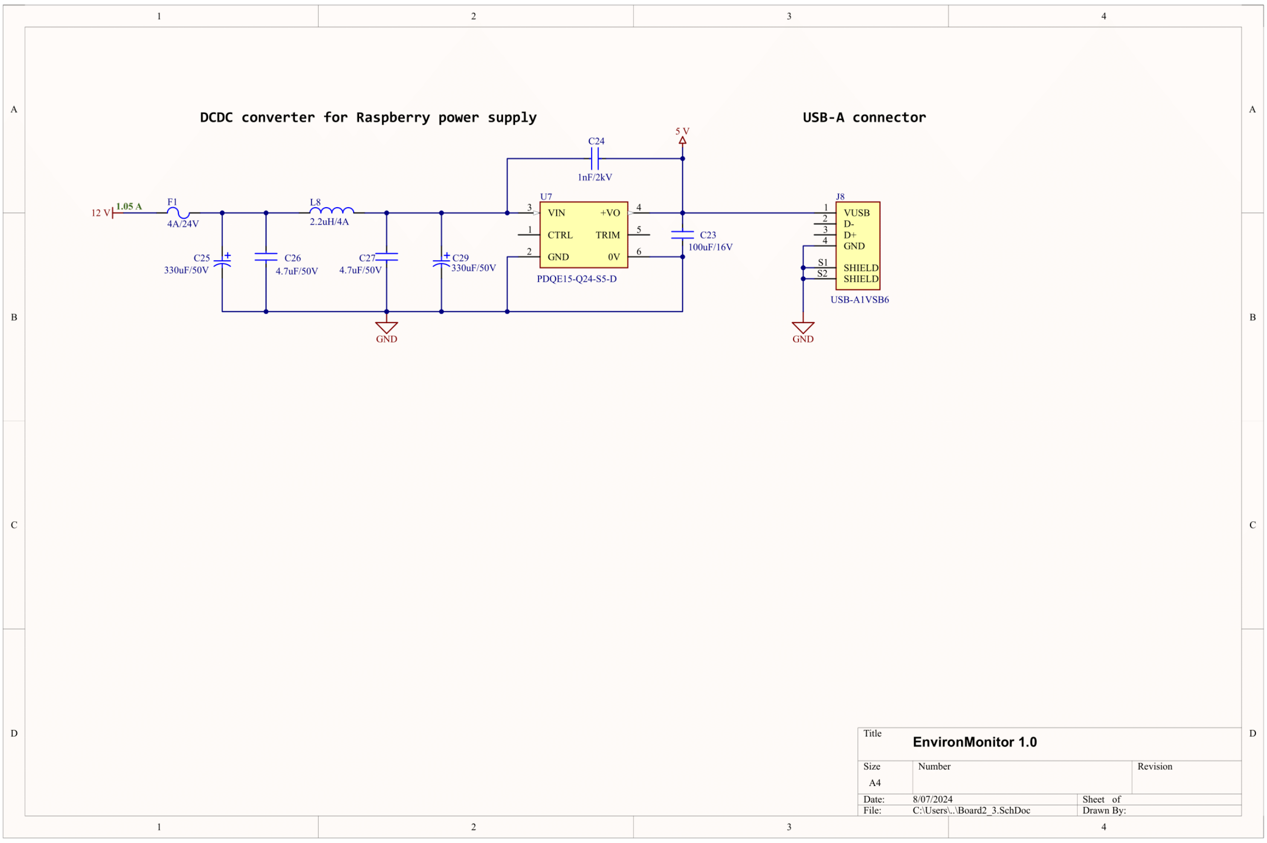

Figure S5. EnvironMonitor third schematic section, intended for the 12 V-to-5 V conversion and the USB-A output used to supply the Raspberry Pi.

Section B

Raspberry Pi acquisition routine

The flowchart of Figure S6 summarises the key points of the implemented firmware logic.

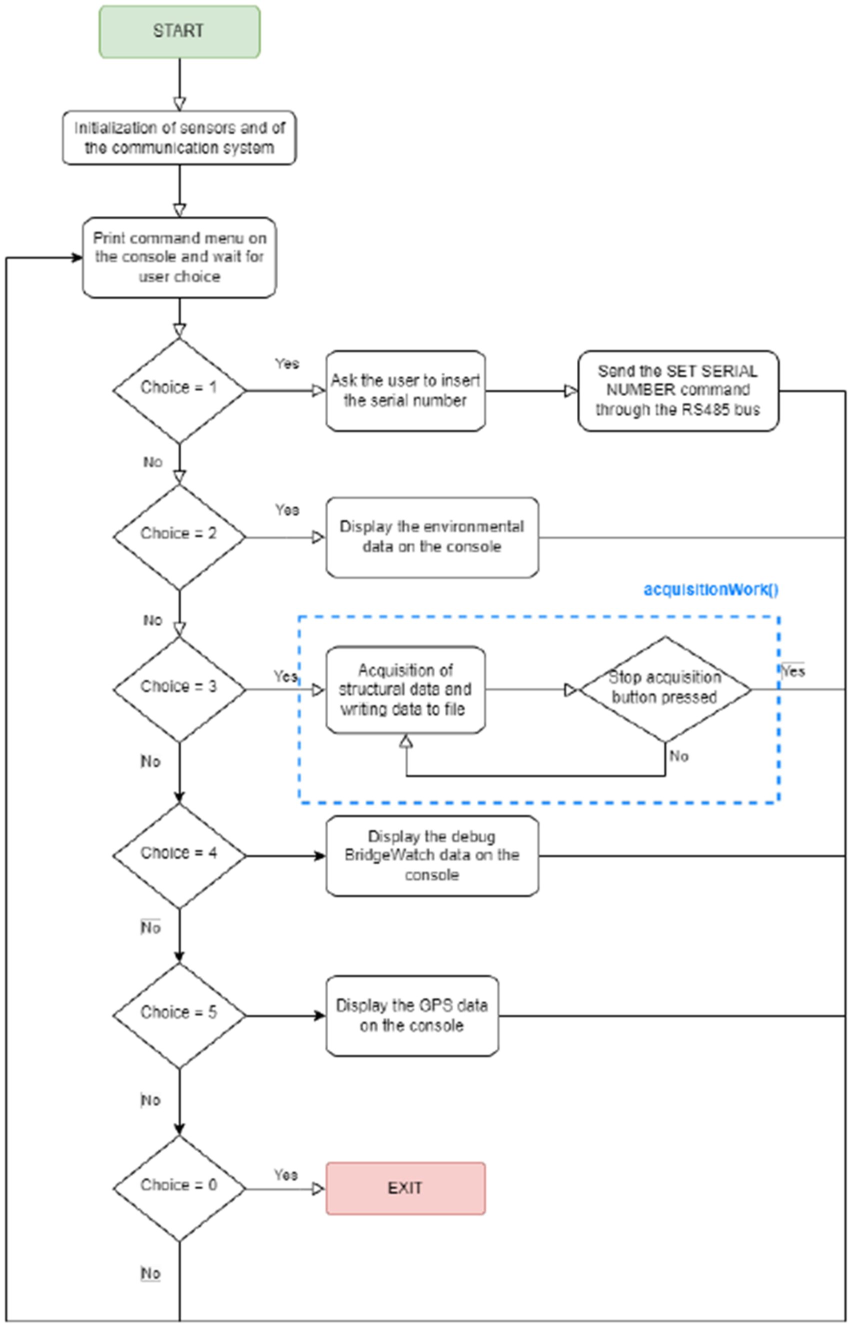

Figure S6. Flowchart of the Raspberry Pi acquisition routine and menu-driven control logic.
